# The ultrafine powder of atractylodis macrocephalae rhizoma improves immune function in naturally aging rats by regulating the PI3K/Akt/NF-κB signaling pathway

**DOI:** 10.3389/fphar.2025.1550357

**Published:** 2025-04-04

**Authors:** Wang Yu, Su Jie, Gao Su, Niu Zhuangwei, Zhou Yiqing, Chen Suhong, Lv Guiyuan

**Affiliations:** College of Pharmaceutical Sciences, Zhejiang Chinese Medical University, Hangzhou, China

**Keywords:** Atractylodis macrocephalae rhizoma, aged, PI3K/Akt/NF-κB, network pharmacology, immunosenescence

## Abstract

**Background:**

The phenomenon of population aging presents a significant global challenge, with the aging population in China steadily increasing. As individuals progress in age, there is a gradual deterioration of human organs and systems, as well as a decline in the immune system, referred to as immunosenescence. Atractylodis macrocephalae rhizoma (BZ) has been historically used in China for its medicinal properties, including gastrointestinal improvement, immunomodulation, anti-aging, antioxidant effects, and anti-tumor effects. Nevertheless, there remains a gap in understanding the pharmacological and molecular mechanisms underlying its anti-immunosenescence effects.

**Methods:**

This study employed UPLC-ESI-MS and network pharmacology to create a network map of BZ ultrafine powder (BZU) and its aging targets. Enrichment analysis was then used to identify the primary mechanistic pathways underlying BZU’s anti-immunosenescence effects. The primary components of BZU were quantitatively analyzed using high-performance liquid chromatography (HPLC). Naturally aging rats were used to examine the effects of different oral doses (0.25, 0.5, and 1 g/kg) of BZU over 5 weeks on aging performance, peripheral blood immunophenotyping and cell count, and splenic lymphocyte proliferation rate. To validate the findings of network pharmacology, quantitative RT-PCR, Western blotting, and immunofluorescence analyses were conducted.

**Results:**

Our analyses demonstrated that BZU improved various indicators of aging in naturally aging rats, such as increasing the number of voluntary activities, enhance grip strength and fatigue resistance, increasing the microcirculatory blood flow and improving hematological levels. The BZU administration enhanced T and B lymphocyte proliferation and significantly improved the lymphocyte-to-T cell subpopulation ratio. It can elevate serum IL-2 and IL-4 levels while reducing IL-6, IFN-γ and TNF-α levels in naturally aging rats. Finally, it increased CD3 protein expression in the spleen while decreasing protein levels of PI3K, p-AKT, IKKα/β, and NF-κB. It also decreased the mRNA expression of *Pik3cg*, *Akt1*, *Pdk1* and *Nf*κ*b1*.

**Conclusion:**

These findings suggest that BZU may enhance lymphocyte proliferation by inhibiting the PI3K/Akt/NF-κB signaling pathway, correcting immune cell imbalances, reducing inflammatory responses, and ultimately enhancing immune function and potentially delaying aging.

## Graphical Abstract

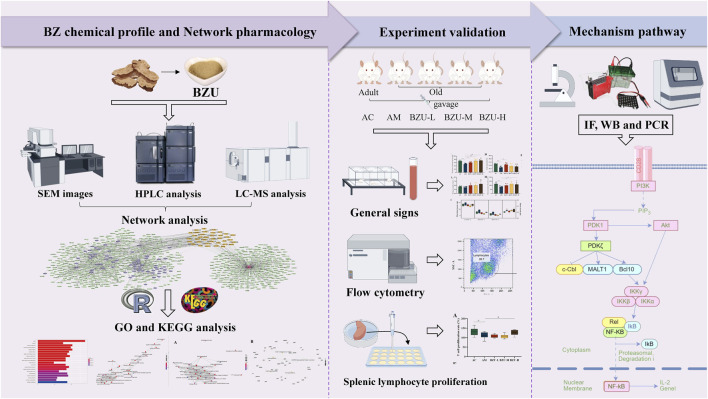

## 1 Introduction

The increasing number and proportion of older individuals represent a significant global demographic challenge. China’s rapidly aging population has emerged as a significant social issue (Wang et al., 2021).The decline in functions related to aging, including reduced immunity, neurasthenia, back and leg pain, and memory loss, significantly affects human health and quality of life (Qiang, 2009). Therefore, improving immunity and delaying aging is an urgent problem.

Aging is a universal phenomenon experienced by all individuals, characterized by the gradual decline of vital essence substances, deterioration of internal organ functions, and structural changes in the body according to Chinese medicine theory (Wanwan and Fusheng, 2019; Mohsen et al., 2020). A report published over 2,000 years ago in the Yellow Emperor’s Classic of Internal Medicine suggested a reduction in spleen and stomach qi (vital energy) would lead to a shorter lifespan (Shiyu, 2017). Therefore, according to Chinese medicine theory, “strengthening the spleen” can slow down the aging process. In Chinese medicine, the “spleen” refers to functions beyond the anatomical organ, involving the spleen, pancreas, and gastrointestinal tract. It is connected to digestion, endocrine, nervous, blood, lymphatic systems, and exocrine glands, all crucial for immunity (Feifei et al., 2018). And in modern medicine, there is also a close relationship between immunity and aging. Aging leads to immunosenescence, causing immune function alterations such as deficiency, autoimmunity, and inflammation imbalance (Matthew et al., 2021).

The aging of the immune system affects both the innate and adaptive immune systems. Natural killer (NK) cells constitute an important effector cell population in the innate immune system. Adaptive immune cells are broadly categorized into T and B lymphocytes. They are distributed throughout the body and, upon antigenic stimulation, activate, divide, proliferate, and initiate specific immune responses. With increasing age, T, B, and NK lymphocytes exhibit signs of senescence, such as disrupted subpopulation cell ratios and dysfunctional cellular responses, leading to impairments in immune response and immune function (Rebeca et al., 2011). In particular, T cells have been the main focus of immunosenescence. (Maria and Guido, 2021). A major division has also been made between T cells that are helpers (Th) and those that are cytotoxic (Tc). Th and Tc cells can be categorized as naïve, memory, or effector T cells, based on their differentiation stage (Snell et al., 2016; Monica et al., 2022). As the body ages, the thymus atrophies, leading to impairments in the development, differentiation, and maturation of T cells. This manifests as a decrease in the number of naive T cells exported from the thymus to the periphery, accompanied by an increase in memory T cells. These cellular changes can reduce the immune response level of elderly individuals to new antigens. The decline of CD8^+^T cells during senescence was more pronounced than that of CD4^+^T cells. The lack of CD28 expression in CD4^+^ and CD8^+^T cells among the elderly is a significant change associated with aging and senescence-related functional processes in human T cells. TH cells can differentiate into subpopulations of helper T cells 1 and 2 (Th1, Th2), Treg cells, and so on under different activation conditions. Th1 and Th2 cells interact with and regulate each other. The increased senescence of peripheral blood T cells leads to higher levels of pro-inflammatory cytokines (IFN-γ, TNF-α, IL-6) and lower levels of anti-inflammatory cytokines (IL-2, IL-4, IL-10) (Cornelia M et al., 2013). CD4^+^CD25^+^Foxp3^+^ Treg cells are the most characteristic Treg cells, capable of inhibiting the proliferation and activation of effector T cells, reducing cytokine secretion, and thereby regulating immune responses to exert immunosuppressive effects. With increasing age, the number of Treg cells increases, and its ability to suppress lymphocyte function by inhibiting lymphocyte activation receptors also enhances (Sanjay et al., 2013). T cells rely on multiple downstream signaling and metabolic pathways to maintain homeostasis and respond appropriately to TCR and cytokine stimulation during activation. Aging disrupts these pathways, most notably by hyperactivating basal signaling pathways in resting T cells, such as an increased basal activation of the PI3K/AKT signaling pathway. NF-κB is a signaling pathway involved in inflammatory responses, and the hyperactivation of the PI3K/AKT pathway can activate nuclear NF-κB, leading to inflammatory reactions that contribute to aging (Carrie et al., 2013).

Atractylodes Macrocephalae Rhizoma (BZ) is the dry rhizome of the Compositae plant *Atractylodes macrocephala* Koidz, which has the effect of invigorate the spleen and Qi, reduce dampness and moisture. BZ, a traditional and precious Chinese herbal medicine in China, has been included in the “List of Items that Can Be Used in Health Foods” by the Ministry of Health, and is expected to be further developed as a food and medicinal substance. BZ can regulate the spleen and stomach, replenish qi and nourish blood, qi and blood transport sufficient, and then enhance the function of the internal organs. It makes the body strong and improves immunity, thus playing a role in prolonging life. To date, there have been many studies on the anti-aging effects of BZ, such as oxidative stress, telomerase, and genetic doctrines, while the functional importance of immunosenescence has been less investigated (Hua et al., 2022). Experimental research has shown that BZ significantly improved learning memory ability, and BZ enhanced the antioxidant capacity of D-galactose-induced senescence model mice (Chunmei et al., 2021). Some studies have found a decrease in plasma malondialdehyde levels, and the rise in superoxide dismutase and total antioxidant activity in mice treated with BZ volatile oil suggests that BZ volatile oil possesses notable antioxidant properties (Yi et al., 2024). Many studies have shown that BZ has the ability to promote the metabolism of organism substances, such as the regulation of body sugar and lipid metabolism, and can prevent and control lipid metabolism disorders, atherosclerosis, and other diseases in the elderly (Qige et al., 2022; Yi et al., 2024). The studies mentioned above thoroughly show that BZ can contribute to delaying the aging process.

In studies, BZ has been proven to enhance and strengthen the body’s immune system. Xiang et al. found that BZ polysaccharides enhance the splenic index, counteract cyclophosphamide-induced immunosuppression in mice, and restore normal immune function (Xuelian et al., 2020). BZ polysaccharide may also mitigate the immunosuppressive effects of cyclophosphamide in geese by preserving the equilibrium between humoral and cellular immunity (Wanyan et al., 2018). Further studies revealed that glycoproteins extracted from BZ are significant immunologically active constituents of BZ extracts, capable of augmenting the induction of protective immune responses in mice (Kyoung et al., 2018). Research has shown that BZ and its active ingredients have an immune enhancing effect.

This paper initially identified the components in BZU using UPLC-ESI-MS analysis, followed by establishing component and disease target network maps through network pharmacology. Subsequently, Gene Ontology (GO) and Kyoto Encyclopedia of Genomes (KEGG) pathway enrichment analyses were conducted to identify the primary mechanistic pathways through which BZU delays aging. The main components of BZU, including Atractylodes I, II, III, and Atractylone, were determined by HPLC. We used naturally aging rats as the old rat model; BZU was administered by gavage for five consecutive weeks at different doses. Subsequently, the impact of BZU on systemic aging manifestations, peripheral blood immunophenotype, and cell counts in aging rats was assessed, along with the proliferation rate of splenic lymphocytes. The study further confirmed the mechanistic pathway through which BZU delays aging by enhancing immunity through immunohistochemistry, Western blotting, and PCR techniques.

## 2 Materials and methods

### 2.1 Data preparation

#### 2.1.1 BZ active ingredient database establishment

UPLC-ESI-MS analysis was performed using a ZenoTOF 7600 mass spectrometer (AB Sciex, California, USA) coupled with a UPLC system (Shimadzu, Rydalmere, Australia).

Chromatographic separation was achieved using a Waters ACQUITY UPLC BEH C18 column (2.1 mm × 50 mm, 1.7 μm) from Milford, MA, USA. Flow rate was 0.3 mL/min, and column temperature was 25°C. Acetonitrile and 0.5% formic acid were used as mobile phases A and B, respectively. A gradient elution in the following manner was applied: 0–8 min, 90–52% B; 8–12 min, 52–50% B; 12–16 min, 50–30% B; 16–18 min, 30–20% B; 18–26 min, 20% B; 26–27 min, 20–90% B; 27–30 min, 90% B, with a 5 μL injection volume.

The positive ion mode was used in the mass spectrometer. The MS parameters were capillary voltage at 3.0 kV (positive ion mode), ion source gas 1 (air) at 55 psi, ion source gas 2 (air) at 55 psi, and curtain gas (N_2_) at 35 psi. The ion spray temperature was 550°C, and the voltage was 5500V. The collision energy was set at 35 V with a spread of 15 V. Analysis of the data was carried out using Analyst TF 1.7 and PeakView 2.1 software (SCIEX).

The experimental data obtained were compared and integrated with the chemical composition of BZ in databases (TCMSP, TCMID, BATMAN-TCM, and Massbank) and literature (PubMed, Embase, CNKI, and Wanfang Database) (Xiaoying et al., 2023; Yafei et al., 2023). The active compound targets were collected from the TCMSP and SymMap databases. Obtaining the compound structural formula of other components in the PubChem database (https://pubchem.ncbi.nlm.nih.gov/) and importing it into SwissTargetPrediction (http://www.swisstargetprediction.ch/index.php) for coupling. The prediction targets were obtained with a probability >0 as the screening conditions.

#### 2.1.2 Collection of drug targets for aging

The genes of targets related to “senescence” or “aging” or “anti-aging” were screened *via* Durgbank (http://www. drugbank. ca/), Malacards (https://www.malacards.org/), TTD (http://db.idrblab.net/ttd/), and OMIM (https://www.ncbi.nlm.nih.gov/omim). After removing the duplicate genes, the common targets of the above keywords are collected as candidates. The BZ and aging targets, including their full names, abbreviations, and gene IDs, were obtained from the Uniprot database (https://www.uniprot.org/).

### 2.2 Drug-compound-target interaction network construction

We utilized Cytoscape 3.9.1 (https://cytoscape.org/) to construct a drug-target-disease map, enabling the analysis of BZ’s active ingredients and their association with aging. Potential target genes for BZ therapy in aging were identified using the Venny 2.1 intersection tool (https://bioinfogp.cnb.csic.es/tools/venny/index.html).

### 2.3 Construction of a PPI protein interaction network

In this study, intersection targets were imported into the STRING database (https://string-db.org/), selecting ‘multiple proteins’ and limiting the species to ‘*Homo sapiens*’. The interrelationship of these potential target genes is imported into Cytoscape 3.9.1, a PPI network is constructed, and core targets are screened out through the calculation for degrees of freedom.

### 2.4 Functional enrichment analysis

Utilize the R packages “*org.Hs.eg.db*” and “*clusterProfiler*” to convert gene symbols to ENTREZID and conduct GO and KEGG enrichment analyses for visualization. The analysis results were filtered to include only those with a *p*-value <0.05 and then sorted by *p*-value and count value for detailed examination.

### 2.5 Preparation and quality control of BZU

#### 2.5.1 Preparation of BZU

Raw herbs were purchased from Zhejiang Intel Pharmaceutical Co., Ltd. (Zhejiang, China). A voucher specimen was made and deposited at the herbarium with voucher specimen number 2010018. The ultrafine powder of BZ was processed by Jinan Dawei Machinery Co., Ltd. and passed through a 300-mesh sieve to obtain drugs with a particle size of <48 μm. A distilled water solution was prepared by dissolving the ultrafine powder in distilled water at 4°C.

#### 2.5.2 Scanning electron microscope observation of BZU

Take an appropriate amount of tissue BZ ultrafine powder and coat it with gold powder on the cross-section surface. Powder morphology was checked using digital light microscopy and a Hitachi SU8010 field emission scanning electron microscope (SEM).

#### 2.5.3 Determination of main components in BZU

The total polysaccharide content of the water extract was determined using the phenol-sulfuric acid method, as outlined in the 2020 edition of the Chinese Pharmacopoeia (Qiuling et al., 2023). HPLC was used to quantify the major components of BZU, such as atractylenolide I, II, III, and atractylone. Analyses of the content of the samples were conducted using an Agilent 2600 series HPLC system (1200, Agilent, Germany) equipped with a diode-array detector (DAD). An Ultimate LP-C18 column (4.6 × 250 mm, 5 μm) was kept at 25°C. The mobile phase comprised 0.01 mol L^−1^ water amine acetate (A) and acetonitrile (B) with a flow rate of 1.0 mL/min. The gradient program was: 0–20 min, 50%–60%B; 20–30 min, 60%–70%B; 30–35 min, 70–100%B; 35–40 min, 100%B; 40–45 min, 100–50%B. The injection volume was 10 μL. The detection wavelengths were 0–23 min, 222 nm; 23–30 min, 278 nm; and 30–45 min, 222 nm, respectively.

### 2.6 Animals and experimental treatment

The research was approved by a university ethics committee (Zhejiang Chinese Medical University Reference ZSLL-2016-118). Adult (4 months) and aged (14–15 months) male SD rats were sourced from Weitong Lihua Limited Company (Zhejiang, China). Rats were maintained under standard conditions (temperature 25°C ± 2°C, humidity 60% ± 5%, 12/12 h day/night cycle) with *ad libitum* access to food and water. The dosing regimen for BZU was determined based on the dosing amount of BZ specified in the Chinese Pharmacopoeia. Young male SD rats were assigned to the young control group (AC, *n* = 6). Aged male SD rats were randomly divided into four groups (*n* = 6 per group): old model group (AM), low-dose BZU group (BZU-L, 0.25 g/kg), medium-dose BZU group (BZU-M, 0.5 g/kg), high-dose BZU group (BZU-H, 1.0 g/kg). The administration was done by oral gavage once a day in the morning for five consecutive weeks.

### 2.7 Behavioral experiment

#### 2.7.1 Autonomic activity test

After 3 weeks of administration, the rats were placed in the autonomous activity instrument (YLS-1A, Jinan Yiyan Technology Development Co., China) in a quiet environment for 5 min after adaptation, and the number of autonomous activities of the rats within 5 min was recorded.

#### 2.7.2 Body temperature test

The body temperature of the rats was measured using rectal probe thermometry (inserting a thermometer into the rectal lumen of the rats) at 3 weeks after administration.

#### 2.7.3 Grip strength test

After 3 weeks of administration, rat forelimb strength was measured using a Rodent Grip Strength Meter (YLS-13A, Jinan Yiyan Technology Development Co., China), which records peak force when grip is lost.

#### 2.7.4 Antifatigue ability test

Evaluation of fatigue resistance in rats by exhaustive swimming time. After 3 weeks of administration, the rats, each loaded with 5% of their body weight in lead skin 1 cm from the tail root, were placed in a swimming tank with 30 cm deep water at 24–26°C. The time counting was started immediately after placing the rats into water, and the end time was recorded when the rat’s head was submerged in water for 5 s without surfacing, and the rats were fished out.

#### 2.7.5 Fecal moisture test

After 3 weeks of administration, freshly excreted stool was collected in sterile tubes, weighed, dried at 60°C for 24 h, and reweighed. Fecal water content (%) was determined using the formula: [(fecal wet weight - fecal dry weight)/fecal wet weight] × 100.

#### 2.7.6 Microcirculatory blood flow intensity test

After 4 weeks of administration, the rats were anesthetized with 4% isoflurane *via* a face mask, and the tail microcirculatory blood flow intensity was measured using a speckle contrast imager (MoorFLPI, Moor Instruments Ltd., Devon, UK).

### 2.8 Immunophenotyping of peripheral blood cells and hematological parameters

#### 2.8.1 Peripheral blood cell count

A 2 mL whole blood sample in an EDTA-treated tube was analyzed for white blood cell (WBC), red blood cell (RBC), hemoglobin (HGB), and blood platelet (PLT) using the routine hematology laboratory method (XT-2000i Automated Hematology Analyzer, Sysmex, Japan).

#### 2.8.2 Immunophenotyping of peripheral blood cell

Peripheral blood immune cell subsets were analyzed *via* flow cytometry as detailed below. We incubated whole blood with surface antibodies for 30 min at 4°C, as detailed in Table 1. To lyse erythrocytes, 1 mL of lysis buffer (BD Pharm Lyse, BD, USA) was added and incubated for 10 min in darkness. Cells were centrifuged at 1100 rpm for 5 min, the supernatant discarded, and then washed with 1 mL of PBS followed by centrifugation at 1100 rpm for 10 min. Cells were washed, centrifuged, resuspended in PBS, and analyzed.

**TABLE 1:** Antibodies used for flowcytometry phenotyping.

Antigen	Flourochrome	Antibody	Clone	Supplier
CD3	FITC	Mouse BALB/c IgM, κ	1F4	BD Pharminge
CD4	BV510	Mouse BALB/c IgG2a, κ	OX-35	BD Pharminge
CD8a	Alexa 647	Mouse BALB/c IgG1, κ	OX-8	BD Pharminge
CD44	BV421	Mouse BALB/c IgG2a, κ	OX-49	BD Pharminge
CD62L	BB700	Armenian Hamster IgG2, λ1	HRL1	BD Pharminge
CD45RA	APC-Cy7	Mouse BALB/c IgG1, κ	OX-33	BD Pharminge
CD28	PE	Mouse BALB/c IgG1, κ	JJ319	BD Pharminge
CD161A	R718	Mouse IgG1, κ	10/78	BD Pharminge

Lymphocyte regions were identified based on their forward scatter (FSC) and side scatter (SSC) characteristics. Non-adhered cells were excluded by FSC-W and FSC-H measurements. The panels define the proportion and total number of major groups as follows: NK cell panel (CD3, CD161a); B cell subset panel (CD3, CD45RA); T cell subset panel (CD3); T helper cells (Th, CD3, CD4); T cytotoxic cell (Tc, CD3, CD8A); naïve, memory, or effector Th and Tc cells panel (CD3, CD4, CD8, CD44, CD62L). Moreover, the expression of CD28 was checked in the subpopulations CD4 and CD8. The expression of Treg cells (CD4^+^CD25^+^Foxp3^+^) was detected.

Peripheral blood lymphocyte and T cell subset immunophenotyping was conducted using multiparameter flow cytometry on a BD LSRFortessa (BD Biosciences). Results were analyzed in FlowJo version 10.6.2 software.

#### 2.8.3 Hematological parameters

Blood was obtained from the abdominal aorta after the rats were anesthetized with ulatan. Plasma viscosity and whole blood viscosity at high, medium, and low shear rates were measured using the capillary method with an FASCO-3010D automatic blood rheology analyzer (FASCO-3010D, Chongqing, China).

### 2.9 Splenic lymphocyte proliferation assay

After the rats were executed, the spleens were removed under sterile conditions. The harvested spleens were promptly placed in 15 mL of ice-cold PBS. Splenocyte single-cell suspensions were prepared by pressing spleens through a 70 μm cell strainer. Red blood cells were lysed using a lysing solution (BD, New York, USA). After two washes with Hank’s solution, the single-cell suspensions were prepared in 500 μL of RPMI 1640 with 10% FBS and 1% penicillin-streptomycin (Gibco, New York, USA). The cells were quantified and adjusted to a working concentration of 1 *×* 10^7^ cells/mL.

CCK8 assays were conducted to evaluate cell proliferation ability. The 100 μL cell suspension was inoculated into a 96-well plate, and splenic T and B lymphocytes were induced to proliferate by the addition of mitogens ConA and LPS, respectively (Kai et al., 2023). The culture was cultured at 37°C in a 5% CO_2_ humidified atmosphere for 72 h. Each well was added with 10 μL CCK8, incubated at 37°C for 2 h, and the OD value at 450 nm was detected by enzymoleter. The proliferation rate was calculated relative to that of the control cells.

### 2.10 Hematoxylin-eosin (HE) staining

The spleen tissue samples were fixed in 10% formaldehyde for over 24 h and subsequently placed in running water overnight. An automated tissue dehydrator (Tissue-TekVIP5Jr, Japan) was used for 8 h to remove excess moisture. The specimen was embedded in paraffin wax and cut into 4 mm-thick sections by a microtome (NanoZoomer S60, Hamamatsu, Japan). After dewaxing and rehydration, the sections were stained with HE for morphological analysis.

### 2.11 Immunohistochemical staining

The spleen paraffin sections were dewaxed using xylene and rehydrated with graded alcohol. Antigens were microwaved in sodium citrate solution (P0083, Beyotime, Shanghai, China) for 15 min and then blocked with 3% hydrogen peroxide (P0260, Beyotime, Shanghai, China) for 15 min. Following a 20-min block using blocking buffer (P0260, Beyotime, Shanghai, China), the sections were incubated overnight at 4°C with anti-CD3 antibody (1:500, 17617-1-AP, Proteintech, Wuhan, China), anti-IKKα/β antibody (1:100, YT2302, ImmunoWay, California, USA), and anti-PDK1 antibody (1:150, ab202468, Abcam, Cambridge, UK). Finally, sections were washed in PBS, developed with DAB solution (19:1 mixture of solution A and B; ZLI-9019, Origene), and counterstained with Hematoxylin. Sections were then observed under a NanoZoomer S60 microscope (Hamamatsu, Japan).

### 2.12 Cytokine measurements

After the last administration, the rats were fasted for 12 h, and blood was collected from the abdominal aorta after anesthesia, and serum was separated (centrifugation at 3,000 rpm for 15 min). Serum levels of IL-2, IL-6, IL-4, IL-10, TNF-α, and IFN-γ were measured using ELISA kits (MultiSciences, Zhejiang, China) following the manufacturer’s instructions.

### 2.13 Western blot analysis

Spleen tissues of rats were powdered in liquid nitrogen, and then the cracking liquid with 1% PMSF was added (Beyotime, Shanghai, China). After lysis for a minimum of 30 min and centrifugation at 12,000 rpm for 10 min at 4°C, protein concentration was measured using a BCA kit (Beyotime, Shanghai, China).

SDS-PAGE gels were prepared using the SDS-PAGE reagent kit (8%). The samples were prerun at 80 V for 30 min, and the samples were fractionated at 120 V for 90 min. The protein was transferred to 0.22 μm PVDF membranes (EMD Millipore) at 200 mA for 120 min. Membranes were blocked for 60 min in 5% nonfat dry milk blocking buffer, then washed in PBST. The membranes were incubated overnight with primary antibodies: anti-PI3K (1:1000, ET1608-70, Huabio, Zhejiang, China), anti-Akt (1:1000, ET1609-47, Huabio, Zhejiang, China), anti-p-Akt (1:500, AF0016, Affinity Biosciences, Texas, USA), anti-CD3 (1:500, 17617-1-AP, Proteintech, Wuhan, China), anti-NF-κB (1:1000, ab76302, Abcam, Cambridge, UK), and anti-β-actin (1:10000, EM21002, Huabio, Zhejiang, China).

Excess primary antibody was washed out, followed by a 2 h incubation with secondary antibody at room temperature and four 5 min washes with PBST. The membranes were incubated with enhanced chemiluminescence reagents (BL520A, Biosharp, Anhui, China) for 1 min at room temperature and then developed in a dark room.

### 2.14 Gene expression analysis by quantitative RT-PCR

Frozen spleen tissue was retrieved from the −80°C freezer and ground into a fine powder using liquid nitrogen. Total RNA was extracted using the SteadyPure Quick RNA Extraction Kit (AG21023, Accurate Biotechnology (Hunan) Co., Ltd., ChangSha, China). Reverse transcription was conducted with the MonScript™ RTIII Super Mix with dsDNase (Two-Step) kit (MR05201, Monad, Suzhou, China). Gene expression levels were measured using the ABI StepOne Plus instrument and the MonAmp™ SYBR ^®^ Green qPCR Mix (High ROX) kit (MQ10301S, Monad), with calculations based on the 2^−ΔΔCT^ method. The primer sequences of genes are listed in Table 2.

**TABLE 2:** Gene primer sequence list.

Rat gene	Sequence (5′to 3′)	Product size
*Pik3cg*-F	CAC​CTC​CGC​AAC​CAA​TCC​TGA​C	130
*Pik3cg*-R	AGC​TCG​AAC​TCT​GTC​TCC​TTC​TGG	
*Pdk1*-F	CTT​AGA​GGG​CTA​CGG​GAC​GGA​TG	117
*Pdk1*-R	TCG​TGG​TTG​GTT​CTG​TAA​TGC​TTC​C	
*Akt1*-F	CAC​AGG​TCG​CTA​CTA​TGC​CAT​GAA​G	95
*Akt1*-R	GCA​GGA​CAC​GGT​TCT​CAG​TAA​GC	
*Nf*κb1-F	TGT​GGT​GGA​GGA​CTT​GCT​GAG​G	138
*Nf*κb1-R	AGT​GCT​GCC​TTG​CTG​TTC​TTG​AG	
*Actin*-F	CAC​GAT​GGA​GGG​GCC​GGA​CTC​ATC	240
*Actin*-R	TAA​AGA​CCT​CTA​TGC​CAA​CAC​AGT	

### 2.15 Statistical analysis

All statistical analyses were conducted using SPSS 26.0, and the data were presented as mean ± standard deviation. A one-way ANOVA was conducted to assess the statistical significance of group differences. When the homogeneity of variance was satisfied, the least significant difference *t*-test was applied. Tambane’s T^2^ method was also employed for analysis. *p* < 0.05 or *p* < 0.01 indicate statistical significance.

## 3 Results

### 3.1 Identification of active ingredients and target selection

The chemical constituents in BZ were analyzed using UPLC-EIS-MS, and a total of 69 active compounds were identified. We list 30 of these major and more abundant components in Table 3.

**TABLE 3:** Identification of compounds in BZ samples by UPLC-EIS-MS.

NO.	tR/min	Molecular formula	Mass	Theoretical mass	Experimental mass	Adduct	MS^2^	Identified constituents
1	0.4	C_6_H_9_N_3_O_2_	155.06948	156.07675	156.07472	+H	110.0727, 83.0596, 56.0483	L-histidine
2	0.47	C_5_H_9_NO_2_	115.06333	116.07061	116.06902	+H	116.0709, 70.0707, 43.0536	L-proline
3	0.83	C_9_H_11_NO_2_	165.07898	166.08626	166.08437	+H	131.0481, 120.0845, 103.0543	L-phenylalanine
4	1.56	C_16_H_18_O_9_	354.09508	355.10236	355.09978	+H	145.0287, 163.0436	Chlorogenic acid
5	3.58	C_10_H_8_O_4_	192.04226	193.04954	193.04754	+H	178.0256, 137.0594, 133.0260	Scopoletol
6	3.7	C_10_H_10_O_4_	194.05791	195.06519	195.06314	+H	177.0525, 145.0269, 117.0338	Ferulic Acid
7	4.5	C_28_H_32_O_15_	608.17412	609.1814	609.17821	+H	463.1194, 301.0702	Neodiosmin
8	4.68	C_13_H_10_O	182.07317	183.08044	183.07601	+H	155.0446, 127.0156, 98.9852	Atractylodin
9	7.08	C_20_H_36_O_7_	388.2461	389.25338	389.25195	+H	371.2419, 232.1680	8,9-epoxy atracolactone
10	8.41	C_16_H_20_O	228.15142	229.15869	229.15639	+H	121.0626, 91.0548, 77.0375	furan sesquiterpene
11	9.4	C_15_H_20_O_3_	248.14124	249.14852	249.14636	+H	175.0747, 163.0780, 135.0435	Atractylenolide Ⅲ
12	10.23	C_16_H_22_O_2_	246.16198	247.16926	247.16697	+H	215.1407, 187.1465, 159.1142	Methyl-atractylenolide II
13	10.34	C_17_H_24_O_3_	276.17254	277.17982	277.17748	+H	259.1674, 235.1757, 217.1562	atractylenolide Ⅳ
14	11.43	C_15_H_20_O_2_	232.14633	233.15361	233.15151	+H	187.1497, 151.0785	Atractylenolide Ⅱ
15	11.93	C_14_H_12_O	196.08882	197.09609	197.09404	+H	128.0643, 115.0524, 91.0541	4,6-Dimethyl dibenzofuran
16	13.85	C_15_H_18_O_2_	230.13068	231.13796	231.13599	+H	185.1317, 157.1004, 145.0999	Atractylenolide Ⅰ
17	14.86	C_15_H_24_	204.1878	205.19508	205.19266	+H	163.1465, 149.0221, 135.1153	beta-Selinene
18	15.01	C_15_H_22_O	218.16707	219.17434	219.17216	+H	123.0811, 95.0856	alpha-Cyperone
19	15.6	C_17_H_22_O_3_	274.15689	275.16417	275.16158	+H	215.1404, 197.1303, 145.0985	3β-acetoxy atractylon
20	16.82	C_6_H_6_O_3_	126.03169	127.03897	127.03703	+H	98.9581, 85.0653, 81.0677	5-Hydroxymethylfurfural
21	16.97	C_17_H_24_O_2_	260.17763	261.18491	261.18222	+H	219.1732, 173.1306, 145.1013	2-(3,7-Dimethyl-octa-2,6-dienyl)-4-methoxy-phenol
22	18.72	C_15_H_22_	202.17215	203.17943	203.17717	+H	147.1186, 133.1025, 119.0851	Atractylenolide Ⅵ
23	18.73	C_6_H_14_N_2_O_2_	146.10553	147.1128	147.11441	+H	105.0676, 91.0532, 41.0368	L-Lysine
24	18.92	C_15_H_20_O	216.15142	217.15869	217.15622	+H	199.1426, 147.1156, 143.0840	Atractylone
25	19.93	C_16_H_30_O	238.22967	239.23694	239.23426	+H	203.1762, 137.1319, 109.0946	Muscone
26	20.04	C_10_H_16_O_2_	168.11503	169.12231	169.12036	+H	151.1100, 133.0997, 123.1159	Trans-geranic acid
27	21.63	C_16_H_32_O_2_	256.24023	257.24751	257.24465	+H	187.1668, 173.1512, 145.1219	Plamitic acid
28	21.92	C_19_H_32_O_2_	292.24023	293.24751	293.24446	+H	243.2104, 237.1812, 183.1332	Methyl Linolenate
29	24.43	C_19_H_34_O_2_	294.25588	295.26316	295.26008	+H	263.2350, 245.2248, 189.1616	Methyl octadeca-9,12-dienoate
30	26.58	C_29_H_48_O	412.37052	413.37779	413.37435	+H	343.0096, 231.0760, 217.1596	Stigmasterol

**FIGURE 1:**
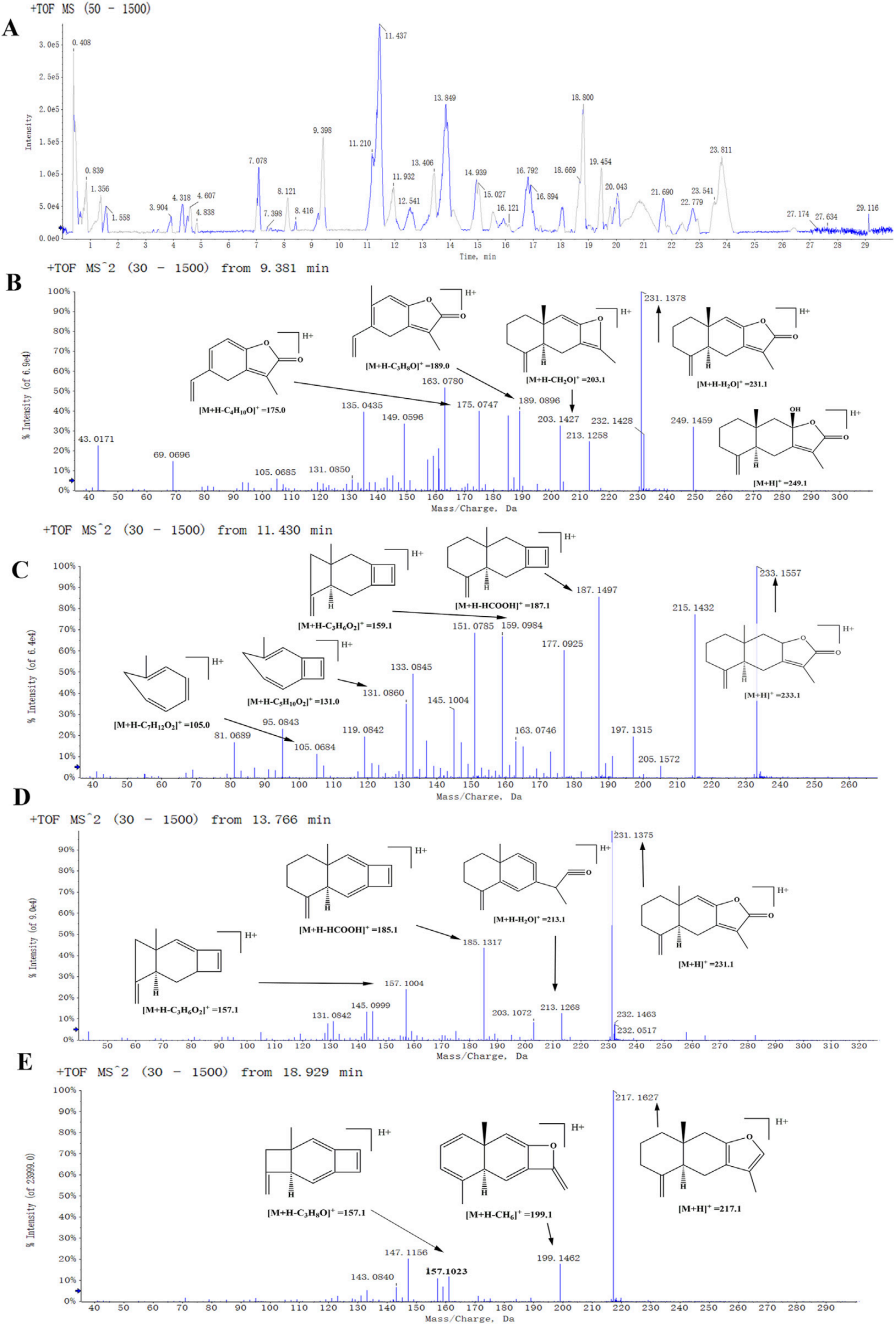
The positive mode detected the mass spectrum of the biomarker. **(A)** A total ion flow chromatogram of BZU. **(B)** Second-order mass spectrum (MS^2^) of the atractylenolide III. **(C)** MS^2^ of the atractylenolide II. **(D)** MS^2^ of the atractylenolide I. **(E)** MS^2^ of the Atractylone.

These components mainly contain sesquiterpenes and lactones, flavonoids, phenylpropanoids, alkynes, polysaccharides, and other components. Among them, sesquiterpenes are the main active ingredients of BZ, mainly including atractylenolide Ⅰ to Ⅶ, atractylenone, and β-eudesmol, etc. The organic acids include chlorogenic acid, ferulic acid, and palmitic acid. The coumarins include 7-hydroxymethylcoumarin and scopoletin. The amino acids include arginine, L-histidine, L-proline, and L-valine. The secondary mass spectra of atractylenolide I, II, III, and atractylone are presented in Figure 1. We have provided the CAS number, SMILE number, and molecule name of all the ingredients in BZ, as well as the Uniprot numbers and abbreviations of the corresponding targets of the ingredients (Supplementary Table S1). A total of 1124 targets corresponding to active ingredients in BZ were collected and predicted. Through the database search of DurgBank, Malacards, TTD, and OMIM, 303 predicted targets about aging were found after deduplication (Supplementary Table S2).

### 3.2 Network construction and PPI protein interaction network

**FIGURE 2:**
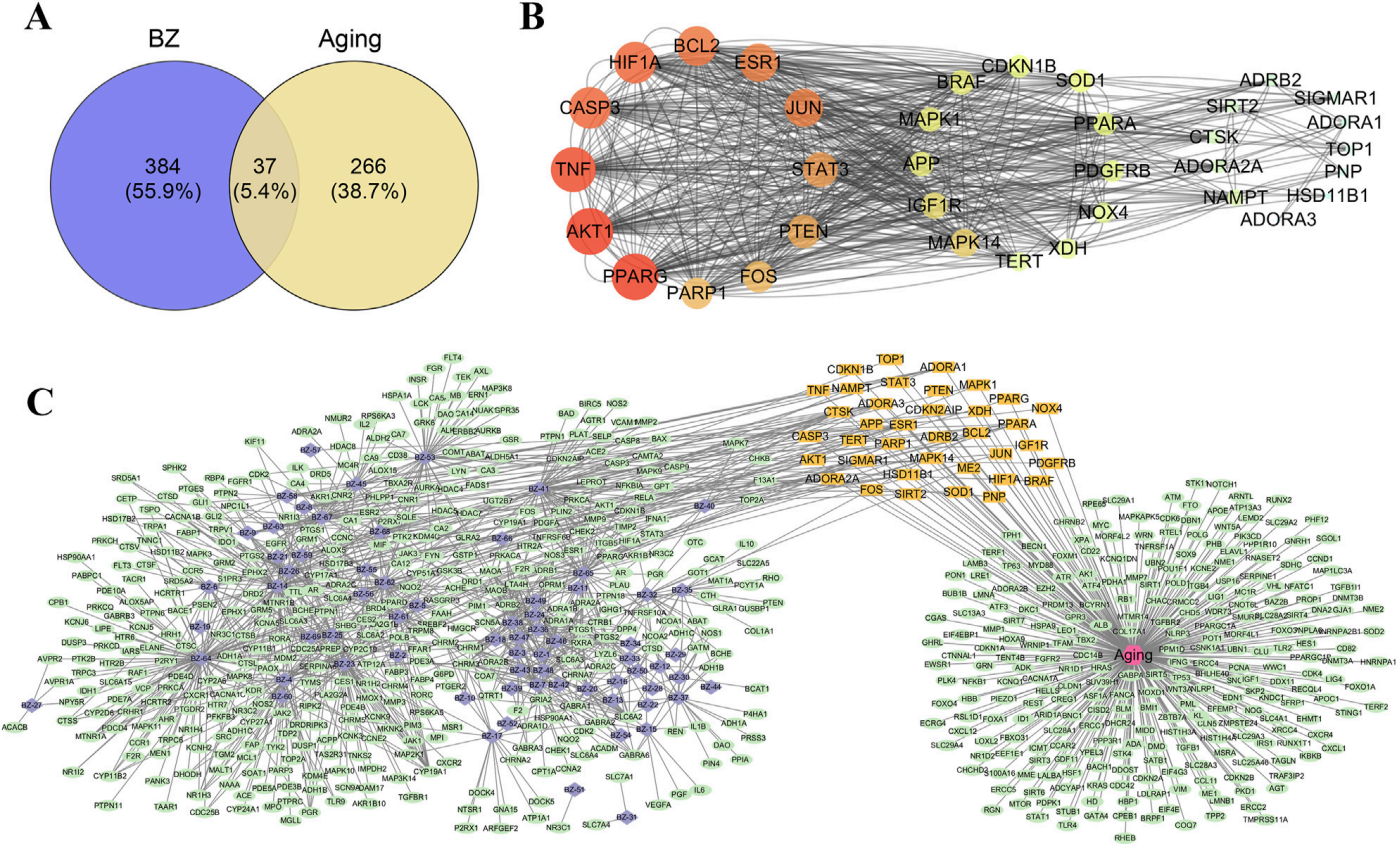
Potential target analysis and PPI network construction. **(A)** The Venny results of potential target genes of BZ therapy for aging. **(B)** Protein-protein interaction network diagram (The corresponding colors in the legend are calculated according to the degrees of freedom). **(C)** Drug-compound–target interaction network diagram (The purple nodes represent the compounds in BZ, the orange nodes represent key targets, and the green nodes represent other targets.).

We import the targets of BZ components and the predicted targets of aging into Cytoscape and Venny for network analysis to obtain the BZ-aging-potential targets gene network (Figure 2). The BZ-aging-potential target gene network comprised 37 nodes and 532 lines (Table 4). Further analysis identified the top 12 targets with significant roles in the network (degree >20): Protein kinase B (AKT1), Poly[ADP-ribose] synthase 1 (PPARG), Tumor necrosis factor receptor superfamily member 10A (TNF), Caspase-3 (CASP3), Hypoxia-inducible factor 1-alpha (HIF1A), Bcl-2-like protein 11 (BCL2), Estrogen receptor (ESR1), Transcription factor AP-1 (JUN), Acute-phase response factor (STAT3), Phosphatidylinositol 3,4,5-trisphosphate 3-phosphatase and dual-specificity protein phosphatase PTEN (PTEN), Proto-oncogene c-Fos (FOS), and PPAR-gamma (PARP1).

**TABLE 4:** 37 potential target genes of BZ therapy for aging.

No.	Symbol	Target	Uniprot	No.	Symbol	Target	Uniprot
1	ADRB2	Beta-2 adrenoceptor	P07550	20	SOD1	Superoxide dismutase [Cu-Zn]	P00441
2	PARP1	Poly [ADP-ribose] synthase 1	P09874	21	CASP3	Caspase-3	P42574
3	CTSK	Cathepsin K	P43235	22	CDKN2AIP	CDKN2AIP N-terminal-like protein	Q96HQ2
4	SIGMAR1	Sigma1-receptor	Q99720	23	CDKN1B	Cyclin-dependent kinase inhibitor 1B	P46527
5	TERT	Telomerase reverse transcriptase	O14746	24	HIF1A	Hypoxia-inducible factor 1-alpha	Q16665
6	ADORA3	Adenosine receptor A3	P0DMS8	25	PPARG	PPAR-gamma	P37231
7	ADORA1	Adenosine receptor A1	P30542	26	FOS	Proto-oncogene c-Fos	P01100
8	ADORA2A	Adenosine receptor A2a	P29274	27	STAT3	Acute-phase response factor	P40763
9	ESR1	Estrogen receptor	P03372	28	TNF	Tumor necrosis factor receptor superfamily member 10A	O00220
10	PPARA	PPAR-alpha	Q07869	29	XDH	Xanthine oxidoreductase	P47989
11	HSD11B1	11-beta-HSD1	P28845	30	NOX4	NADPH oxidase 4	Q9NPH5
12	AKT1	Protein kinase B	P31749	31	PDGFRB	Platelet-derived growth factor receptor beta	P09619
13	TOP1	DNA topoisomerase 1	P11387	32	IGF1R	Insulin-like growth factor 1 receptor	P08069
14	MAPK14	Mitogen-activated protein kinase 14	Q16539	33	BRAF	Serine/threonine-protein kinase B-raf	P15056
15	MAPK1	Mitogen-activated protein kinase 13	O15264	34	PNP	Purine nucleoside phosphorylase	P00491
16	ME2	NAD-dependent malic enzyme, mitochondrial	P23368	35	APP	Amyloid-beta precursor protein	P05067
17	BCL2	Bcl-2-like protein 11	O43521	36	NAMPT	Nicotinamide phosphoribosyltransferase	P43490
18	PTEN	Phosphatase and tensin homolog	P60484	37	SIRT2	NAD-dependent protein deacetylase sirtuin-2	Q8IXJ6
19	JUN	Transcription factor AP-1	P05412				

### 3.3 GO and KEGG pathway enrichment analysis

**FIGURE 3:**
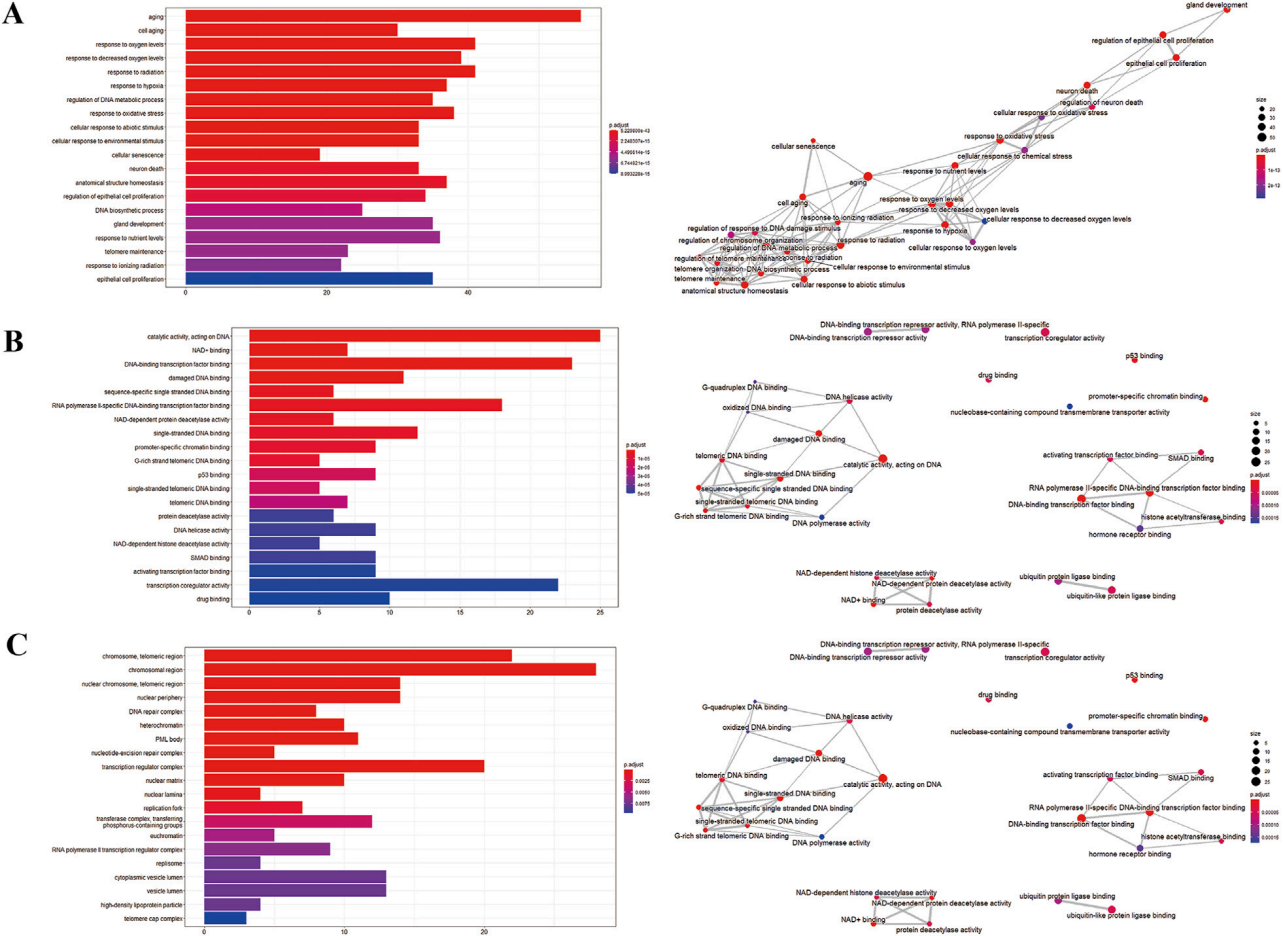
GO enrichment analysis of potential genes. **(A)** Biological process analysis. **(B)** Molecular function analysis. **(C)** Cell component analysis.

We imputed 37 targets into the R language, enriched and analyzed the GO and KEGG pathways. Annotation of GO function enrichment is based on three aspects: biological process (BP), molecular function (MF), and cellular component (CC) (Supplementary Table S3). GO enrichment analysis identified 1990 biological process functions (*p* < 0.05), encompassing aging, cell aging, responses to oxygen and decreased oxygen levels, radiation, hypoxia, regulation of DNA metabolic processes, and oxidative stress. Scatterplots were generated for the top 20 enriched gene biological function catalogs and the top five most significant biological function enriched targets (Figure 3). GO analyses identified 120 enriched molecular function GO terms, such as catalytic activity on DNA, NAD+ binding, DNA-binding transcription factor binding, damaged DNA binding, sequence-specific single-stranded DNA binding, and RNA polymerase II-specific DNA-binding transcription factor binding. 52 GO terms in the cellular component were enriched, including chromosome, telomeric region, chromosomal region, nuclear chromosome, telomeric region, nuclear periphery, DNA repair complex, etc. The most significant genes in these pathways involved were TP53, JUN, HIF1A, STAT3, SIRT1, CDKN2A, FOXO3, etc.

**FIGURE 4:**
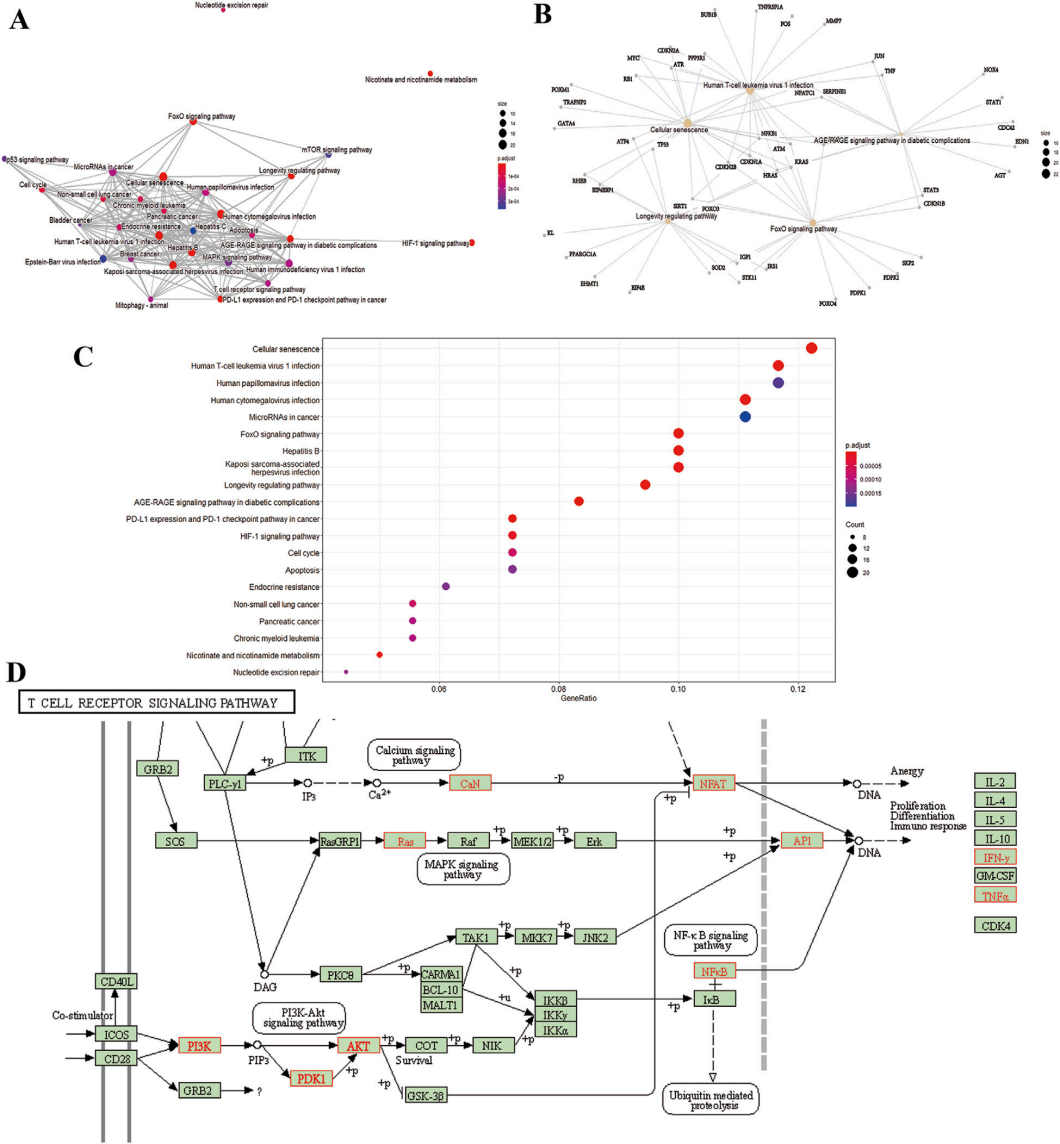
KEGG pathway analysis. **(A)** Interaction network of pathways. **(B)** Target-pathway network. **(C)** Top 20 genes of pathway enrichment. **(D)** The T cell receptor signaling pathway of potential target genes of BZ in aging. Arrows indicate upstream and downstream relationships between genes. The red is a BZ target gene in the network.

To gain insight into gene regulatory networks in BZ therapy for aging, a total of 102 KEGG signaling pathways were enriched (Supplementary Table S4). The top 20 highly enriched KEGG pathways are presented (Figures 4A–C). The KEGG analysis identified pathways including Cellular senescence, MAPK, PI3K-Akt, FoxO, mTOR, and T cell receptor signaling, etc. Figure 4D highlights the primary targets in the T cell receptor signaling pathway.

### 3.4 Quantitative analysis of components in BZU

**FIGURE 5:**
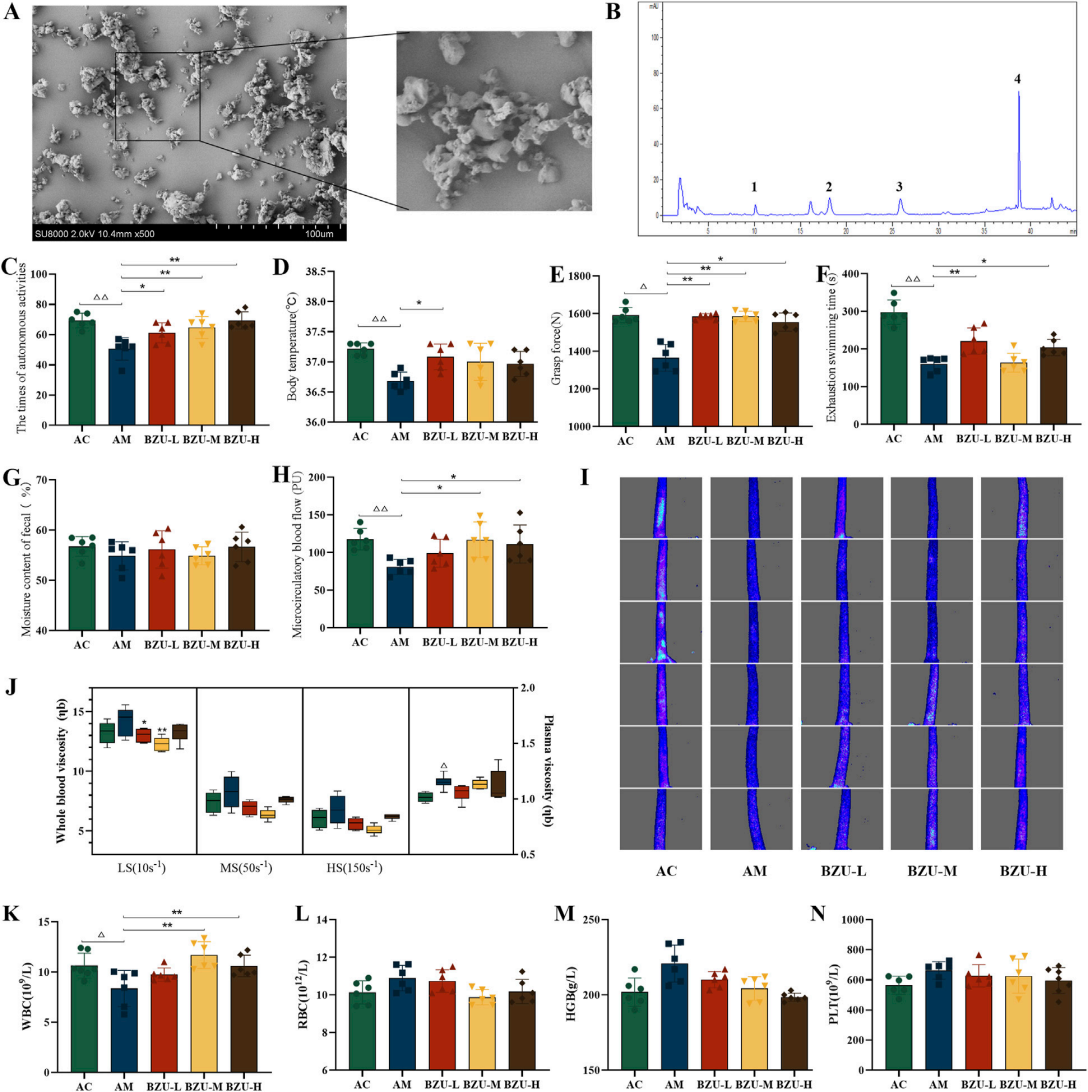
BZU content determination and behavioral results. **(A)** The SEM micrograph images of BZU. **(B)** HPLC chromatogram of the content of the main components of BZU (Peaks 1,2,3,4 are atractylenolide III, II, I and atractylone respectively.). Behavioral experiment: **(C)** the times of autonomous activity, **(D)** the body temperature, **(E)** the grasp strength, **(F)** the exhaustive swimming time, **(G)** the moisture content of fecal, **(H)** the microcirculatory blood flow and **(I)** densitometry of microcirculatory blood flow in the rat tail. **(J)** Changes of hemorheology indexes. Peripheral blood measurements for **(K)** WBC count, **(L)** RBC count, **(M)** HGB count and **(N)** PLT count. Statistical significance of differences between groups is depicted on the graphs. Data were shown as mean ± SD (Each group *n* = 6). AC, Young control group; AM, Old model group; BZU-L, low-dose BZU group; BZU-M, medium-dose BZU group; BZU-H, high-dose BZU group. ^△^ *p* < 0.05, ^△△^ *p* < 0.01. vs. control; *p < 0.05, **p < 0.01. vs. model.

As can be noticed in the SEM micrograph (Figure 5A), the *BZ*U is incomplete with large fragmentation and uniformity in particle size. The surfaces of the particles are rough, and the powders agglomerate with each other.

The phenol-sulfuric acid method determined the total polysaccharide content of BZU to be 4.76%. In addition, the contents of atractylenolide I, II, III, and atractylone in BZU, determined by HPLC, were 0.2962, 0.3970, 0.2316, and 1.7089 mg/g, respectively. HPLC chromatograms of the BZU are depicted in Figure 5B.

### 3.5 Effects of BZU on general signs

As depicted in Figures 5C–I, compared to the AC group, the times of autonomous activity, the body temperature, the grasping strength, the times of exhaustive swimming, and the microcirculatory blood flow of the AM group were significantly decreased (*p* < 0.05, *p* < 0.01). After 3 weeks of administration, all BZU groups exhibited a significant increase in autonomous activity, and the BZU-L group showed a significant rise in body temperature compared to the AM group (*p* < 0.05, *p* < 0.01) (Figures 5C,D). All BZU groups exhibited a significant increase in grasp strength compared to the AM group (*p* < 0.05, *p* < 0.01) (Figure 5E). The BZU-L and BZU-H groups showed a significant rise in exhaustive swimming time compared to the AM group (*p* < 0.05, *p* < 0.01) (Figure 5F). However, no significant differences were observed in fecal water content (*p* > 0.05, *p* < 0.01) (Figure 5G). Microcirculatory blood flow significantly increased in the BZU-M and BZU-H groups compared to the AM group (*p* < 0.05, *p* < 0.01) (Figure 5H). As Figure 5I shows, at a glance, the change in intensity of microcirculatory blood flow in the tail of each group of rats.

### 3.6 Effect of BZU on hematological indices

Figure 5J illustrates that both whole blood and plasma viscosity were significantly elevated in the AM group compared to the AC group, with plasma viscosity showing a notable difference (*p* < 0.05). Post-intervention, whole blood viscosity at low shear rates was significantly reduced in both the BZU-L and BZU-M groups compared to the AM group (*p* < 0.05, *p* < 0.01).

The AM group exhibited a significant decrease in WBC levels compared to the AC group (*p* < 0.05), while the levels of RBC, HGB, and PLT showed an increasing trend without reaching statistical significance (*p* > 0.05). After 3 weeks of administration, the BZU-M and BZU-L groups exhibited a significant increase in WBC levels compared to the AM group (Figure 5K). The RBC, HGB, and PLT levels showed a downward trend, but no significant differences were found (*p* > 0.05) (Figures 5L–N).

### 3.7 Effect of BZU on peripheral blood immunophenotyping ratios

**FIGURE 6:**
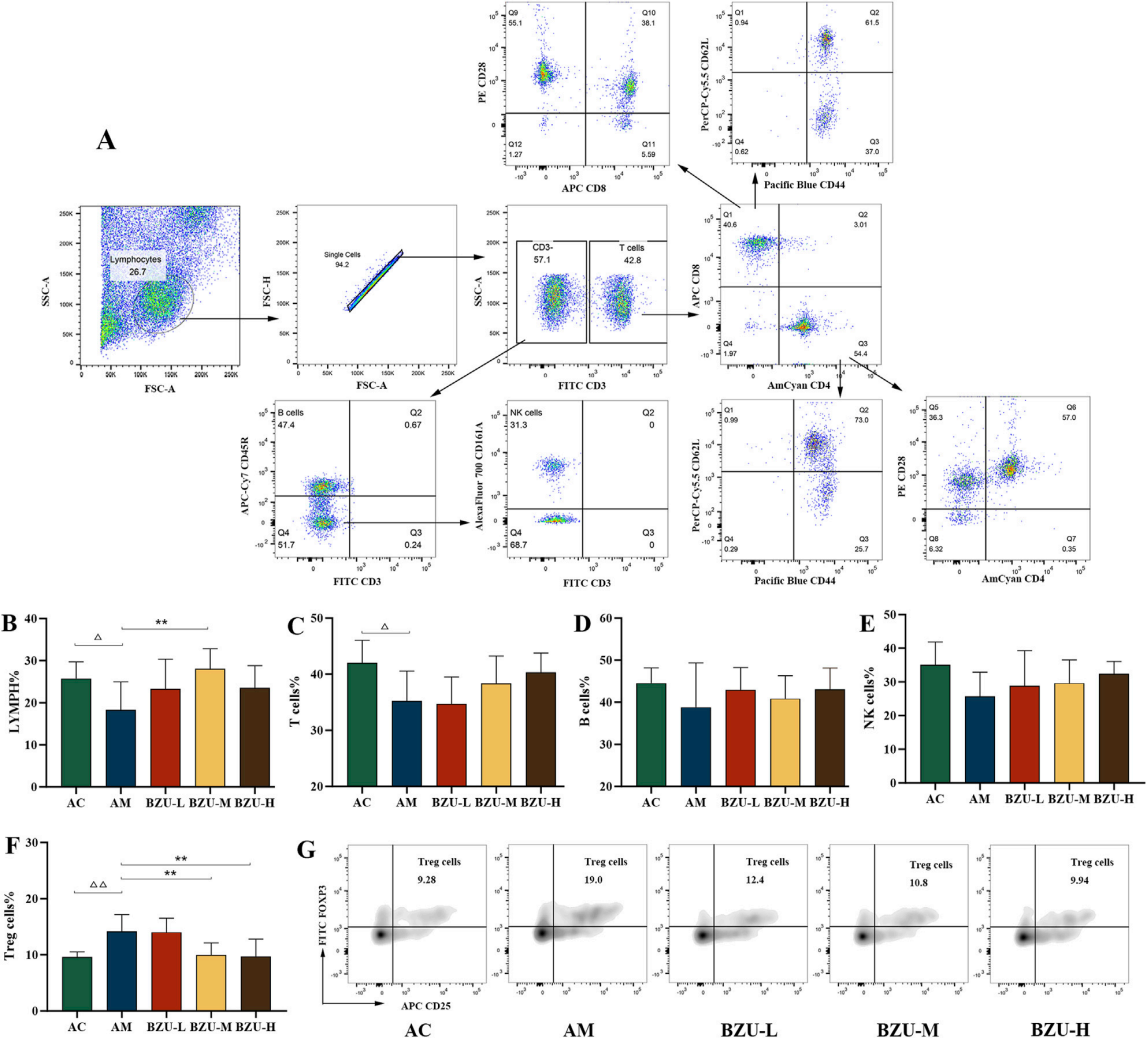
Phenotype analysis of lymphocyte subpopulations by flow cytometry. **(A)** Flow cytogram circle gate logic diagram. **(B)** Lymphopenia, **(C)** T cells (CD3^+^), **(D)** B cells (CD3^−^CD45RA^+^), **(E)** NK cells (CD3^−^CD161a^+^), **(F)** Treg cells (CD4^+^CD25^+^Foxp3^+^) and **(G)** Flow densitogram of Treg cells. Statistical significance of differences between groups is depicted on the graphs. Data were shown as mean ± SD (Each group *n* = 6). AC, Young control group; AM, Old model group; BZU-L, low-dose BZU group; BZU-M, medium-dose BZU group; BZU-H, high-dose BZU group. ^△^ *p* < 0.05, ^△△^ *p* < 0.01. vs. control; *p < 0.05, **p < 0.01. vs. model.

The logic diagram of flow cytometry gating of peripheral blood immune cells is shown in Figure 6A. Figures 6B,C show that compared with the AC group, the proportion of lymphocytes and T cells in the AM group significantly decreased (*P* < 0.05). There was a trend toward a decrease in the percentage of B and NK cells in the AM group, but there was no significant difference (*p* > 0.05) (Figures 6D,E). While the proportion of Treg cells in the AM group was significantly higher (*p* < 0.05) (Figures 6F,G). After 3 weeks of oral gavage administration of BZU, the lymphocyte percentage significantly increased in the BZU-M group compared to the AM group (*p* < 0.01). T, B, and NK lymphocyte levels increased in the treatment group, but the change was not statistically significant (*p* > 0.05). The proportion of Treg cells in the BZU-M and BZU-H groups was significantly reduced.

**FIGURE 7:**
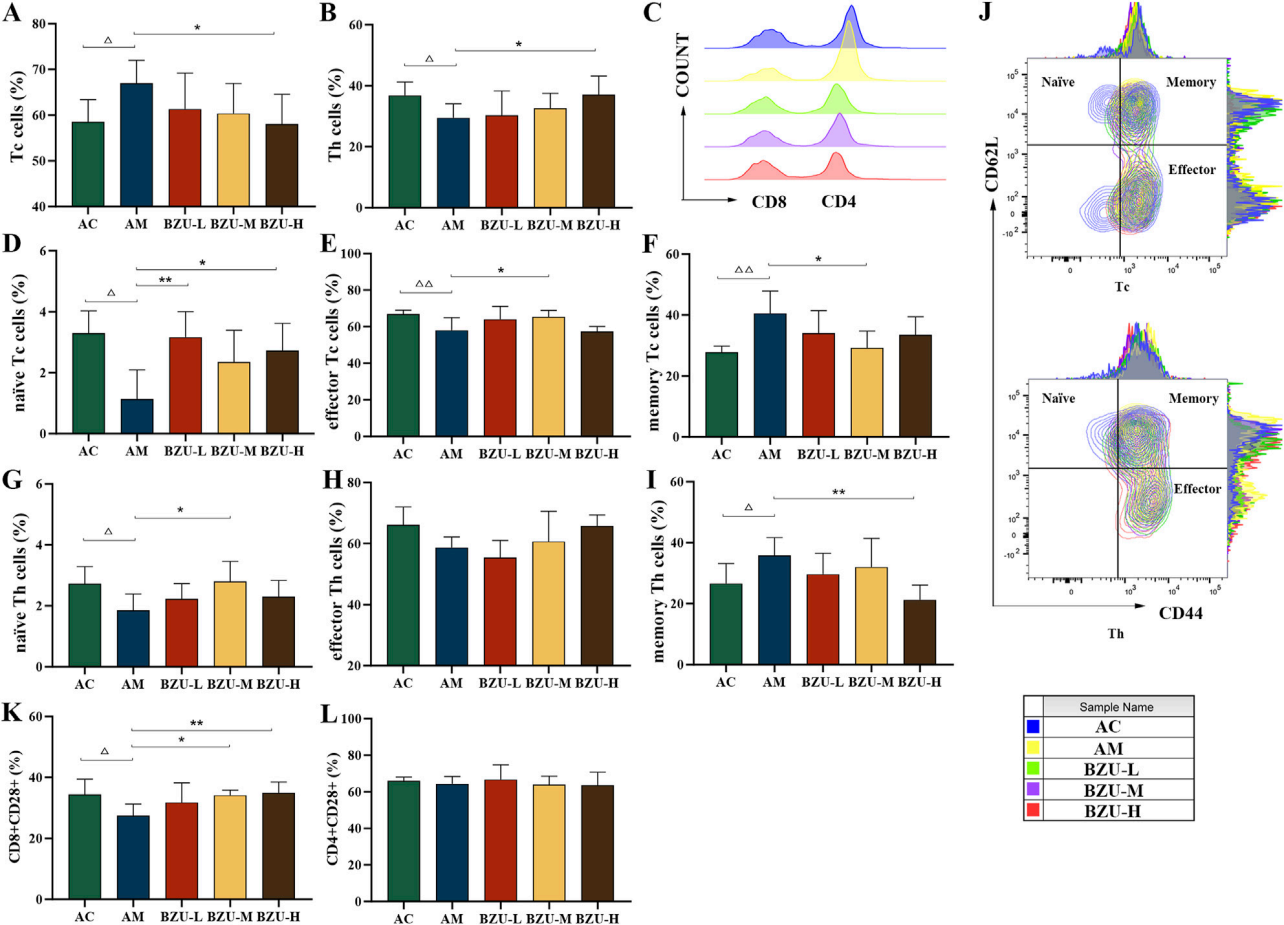
Phenotype analysis of T cell sub-populations by flow cytometry. **(A)** Tc cells (CD3^+^CD8^+^), **(B)** Th cells (CD3^+^CD4^+^), **(C)** Histogram of CD4/CD8 cells, **(D)** naïve Tc cells (CD3^+^CD8^+^CD44^−^CD62^+^), **(E)** effector Tc cells (CD3^+^CD8^+^CD44^+^CD62^−^), **(F)** memory Tc cells (CD3^+^CD8^+^CD44^+^CD62^+^), **(G)** naïve Th cells (CD3^+^CD4^+^CD44^−^CD62^+^), **(H)** effector Th cells (CD3^+^CD4^+^CD44^+^CD62^−^), **(I)** memory Th cells (CD3^+^CD4^+^CD44^+^CD62^+^), **(J)** Contour map of T-cell subpopulations, **(K)** CD8^+^CD28^+^, **(L)** CD4^+^CD28^+^. Statistical significance of differences between groups is depicted on the graphs. Data were shown as mean ± SD (Each group *n* = 6). AC, Young control group; AM, Old model group; BZU-L, low-dose BZU group; BZU-M, medium-dose BZU group; BZU-H, high-dose BZU group. ^△^ *p* < 0.05, ^△△^ *p* < 0.01. vs. control; *p < 0.05, **p < 0.01. vs. model.

Figure 7 shows that compared with the AC group, the proportion of Tc cells in the AM group significantly increased, while the proportion of Th cells significantly decreased (*p* < 0.05). In addition, there was a significant difference in the proportion of naïve T, memory T and effector T cells between the two groups. The proportions of memory of Th and Tc cells in the AM group were significantly increased, while the proportions of naïve of Th and Tc cells, and effector Tc cells were significantly decreased (*p* < 0.05, *p* < 0.01). The AM group showed a significant decrease in the proportion of CD8^+^CD28^+^ subgroups, while there was no statistically significant difference in the proportion of CD4^+^CD28^+^ subgroups (*p* < 0.05).

The BZU-H group significantly increased the percentage of Th and naïve Tc cells and decreased the percentage of Tc and memory Th cells (*p* < 0.05, *p* < 0.01) (Figures 7A–I). The BZU-M group significantly increased the percentage of effector Tc cells and naïve Th cells and decreased the percentage of memory Tc cells (*p* < 0.05, *p* < 0.01). The BZU-L group significantly increased the percentage of naïve Tc cells (*p* < 0.01). As shown in Figure 7J, a contour plot of the T-cell subpopulations, the distribution of cell proportions can be visualized. The CD8^+^CD28^+^ subsets were significantly elevated in the BZU-M and BZU-H groups (*p* < 0.05, *p* < 0.01) (Figure 7K). BZU had no significant effect on CD4^+^CD28^+^ cells (Figure 7L).

### 3.8 Effect of BZU on the proliferation rate of spleen lymphocytes

**FIGURE 8:**
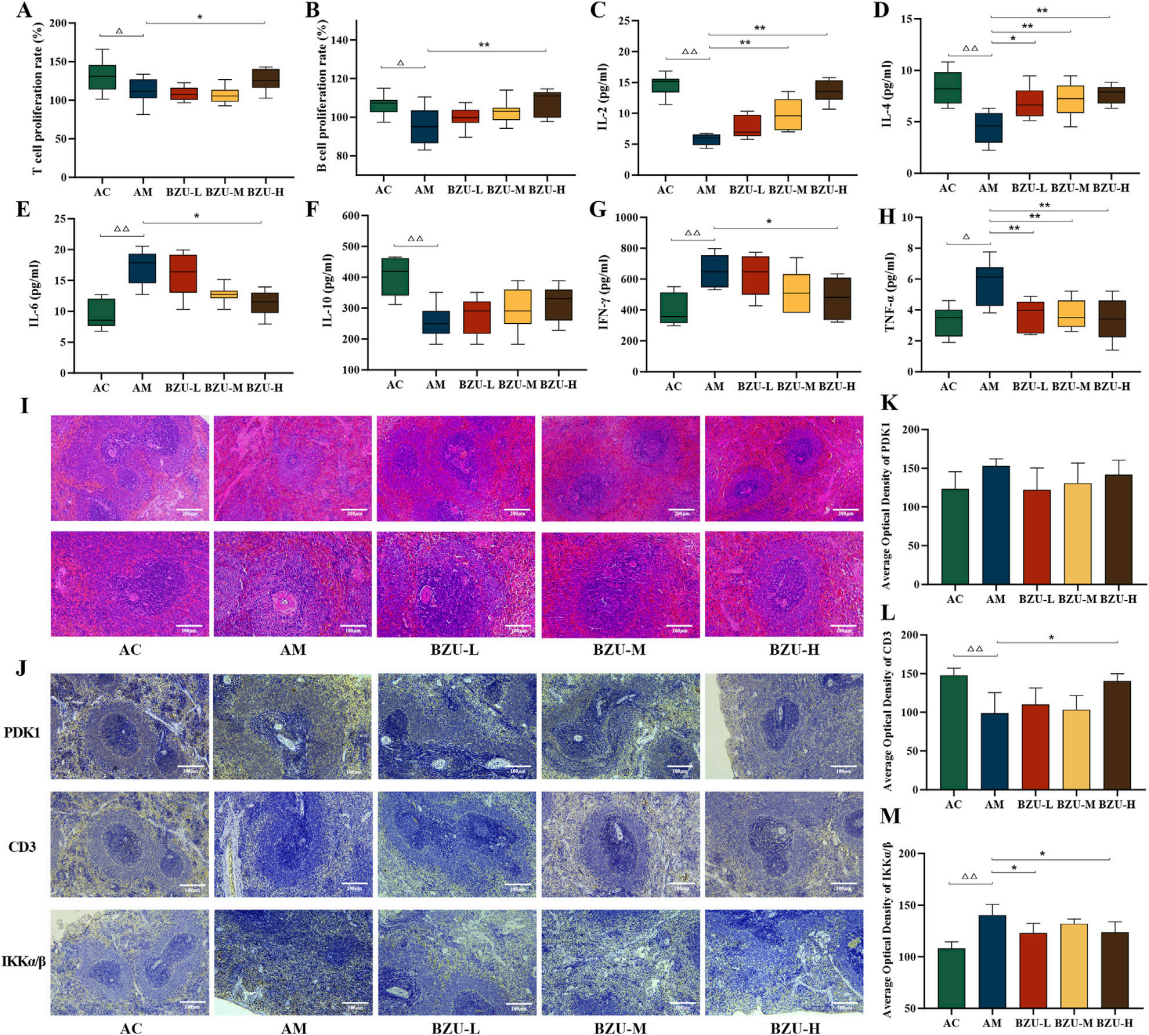
Lymphocyte proliferation rate, immunohistochemical expression and serum levels of inflammatory factors in the spleen. **(A)** T-lymphocyte proliferation rate. **(B)** B-lymphocyte proliferation rate. The content of **(C)** IL-2, **(D)** IL-4, **(E)** IL-6, **(F)** IL-10, **(G)** IFN-γ and **(H)** TNF-α in serums. Data were shown as mean ± SD (Each group *n* = 6). **(I)** Histopathology observations of spleen tissue (10×, 20×). **(J)** Immunohistochemistry plot of PDK1, CD3 and IKKα/β (20×). The immunohistochemical quantitative statistics of **(K)** PDK1, **(L)** CD3, and **(M)** IKKα/β. Data were shown as mean ± SD (Each group *n* = 3). Statistical significance of differences between groups is depicted on the graphs. AC: Young control group; AM: Old model group; BZU-L: low-dose BZU group; BZU-M, medium-dose BZU group; BZU-H, high-dose BZU group. ^△^ *p* < 0.05, ^△△^ *p* < 0.01. vs. control; *p < 0.05, **p < 0.01. vs. model.

Compared with the AC group, the proliferation ability of T cells and B cells in the spleen of rats in the AM group was significantly reduced, as shown in Figures 8A,B (*p* < 0.05). The BZU-H group exhibited a significantly higher proliferation rate of splenic T and B cells in rats compared to the AM group (*p* < 0.05, *p* < 0.01).

### 3.9 Changes of serum IL-2, IL-4, IL-6, IL-10, TNF-α, and IFN-γ levels

Serum levels of IL-2, IL-4, and IL-10 were significantly lower, whereas IL-6 and TNF-α levels were significantly higher in the AM group compared to the AC group (*p* < 0.05, *p* < 0.01) (Figures 8C–H).

After drug administration, serum IL-2 levels significantly increased in both BZU-M and BZU-H groups (*p* < 0.01). The serum levels of IL-6 and IFN-γ in the BZU-H group were significantly reduced (*p* < 0.05). All BZU administration groups significantly increased IL-4 and decreased serum TNF-α levels (*p* < 0.05, *p* < 0.01).

### 3.10 Effect of BZU on spleen tissue morphology

Figure 8I’s HE staining pathological results revealed that the spleens of AC group rats exhibited a clear structure, closely arranged lymphocytes, and well-defined red and white pulp with distinct edges. The spleens in the AM group exhibited disrupted structures with indistinct margins between the white and red pulps. The white pulp appeared blurred and reduced in size, with a normal but sparse lymphocyte population. Compared with the AM group, the white pulp of the spleen of rats in the BZU-M and BZU-H groups was significantly enlarged, the red and white pulp structures were clearly demarcated, and the histopathological changes of the spleen were all significantly improved.

### 3.11 Localization and expression levels of PDK1, CD3, and IKKα/β proteins in rat spleen tissue

As shown in Figure 8J, CD3^+^T cells were predominantly distributed in the white pulp. In the AM group, the number of periarterial lymphatic sheath cells composed of T lymphocytes was reduced. The proteins IKKα/β and PDK1 exhibit predominant expression within the red pulp and cortical structures of splenic tissue. Compared with the AC group, the expression level of IKKα/β protein was significantly increased, and CD3 expression was significantly decreased in the AM group (*p* < 0.01). Post drug administration, CD3 protein expression significantly increased in the BZU-H group, while IKKα/β protein expression significantly decreased in the BZU-L and BZU-H groups (*p* < 0.05).

### 3.12 Levels of PI3K, AKT, p-AKT, NF-κB, and CD3 as determined by western blotting

**FIGURE 9:**
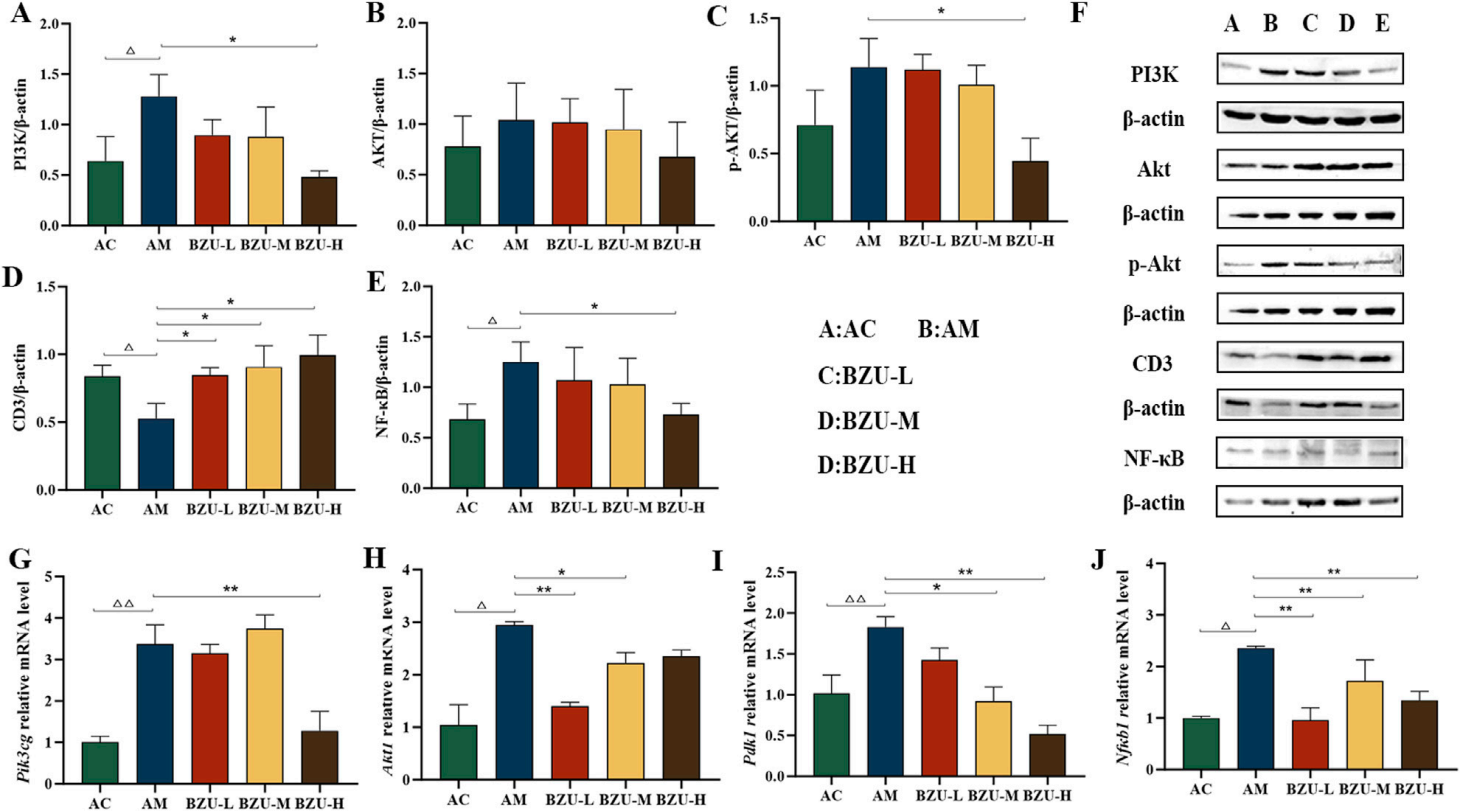
BZU regulates the expression of PI3K/Akt/NF-κB pathway in the spleen. The protein expression of **(A)** PI3K, **(B)**AKT, **(C)** p-AKT, **(D)** CD3 and **(E)** NF-κB. **(F)** WB images of PI3K, AKT, p-AKT, CD3 and NF-κB protein expression. The mRNA expression of **(G)** *Pik3cg*, **(H)** *Akt1*, **(I)** *Pdk1* and **(J)** *Nf*κ*b1*. Statistical significance of differences between groups is depicted on the graphs. Data were shown as mean ± SD (Each group *n* = 3). AC, Young control group; AM, Old model group; BZU-L, low-dose BZU group; BZU-M, medium-dose BZU group; BZU-H, high-dose BZU group. ^△^ *p* < 0.05, ^△△^ *p* < 0.01. vs. control; *p < 0.05, **p < 0.01. vs. model.

Figures 9A–E show that compared with the AC group, the expression levels of PI3K and NF-κB proteins in the spleen tissue of the AM group were significantly increased, while the expression level of CD3 protein was significantly decreased (*p* < 0.05). The expression of p-AKT protein showed an upward trend, but there was no significant difference (*p* > 0.05). After administration, the protein expression of PI3K, p-AKT, and NF-κB in the spleen tissue of rats in the BZU-H group was significantly reduced. The expression of CD3 proteins was significantly increased in all administration groups (*p* < 0.05). The immunoblot band diagram is shown in Figure 9F.

### 3.13 mRNA expression levels of *Pik3cg*, *Akt1*, *Pdk1* and *Nfκb1* in tissues as determined by qRT-PCR

The AM group exhibited significantly elevated mRNA expression levels of *Pik3cg*, *Akt1*, *Pdk1* and *Nf*κ*b1* compared to the AC group (*p* < 0.01). The BZU-H group exhibited significantly reduced mRNA expression levels of *Pik3cg* compared to the AM group (*p* < 0.01). Additionally, the BZU-M and BZU-H groups reduced *Pdk1* mRNA expression, while the BZU-L and BZU-M groups reduced *Akt1* mRNA expression (*p* < 0.05, *p* < 0.01). All BZU administration groups administration groups significantly reduced *Nf*κ*b1* mRNA expression. (Figures 9G–J).

## 4 Discussion

According to relevant statistics, China’s elderly demographic, defined as individuals aged 65 and older, constituted 11.4% of the nation’s total population by the conclusion of 2017, amounting to 158 million individuals (Qidi et al., 2021). Furthermore, projections indicate that between 2000 and 2055, the rate of population aging in China is expected to surge from 10% to 34%, surpassing the global average growth rate. A 2019 study in The Lancet identified stroke, ischemic heart disease, lung cancer, and Alzheimer’s disease as leading causes of mortality in China, highlighting the serious social issue of accelerated population aging (Maigeng et al., 2017). Therefore, slowing down the aging process has become an important scientific problem that needs to be solved.

Aging results in a gradual decline in physiological and organ functions, weakening physical and mental states. This decline may manifest in symptoms such as neurasthenia, cold intolerance, decreased physical strength, memory impairment, microcirculation issues, and back and leg pain (Elsa et al., 2017). Therefore, this test evaluated the effect of BZU by observing these apparent indicators of aging. We evaluated autonomous activity times, body temperature, grip strength, swimming endurance, and microcirculatory blood flow in aging rats to examine the impact of BZU on mental mood, metabolic function, physical performance, and microcirculation. It was found that BZU could increase the times of autonomous activity, raise body temperature, enhance grip strength and fatigue resistance, and increase the microcirculatory blood flow of naturally aging rats. This suggests that BZU can modulate various functional impairments and improve biological indicators of aging in naturally aging rats.

In addition, as humans age, the bone marrow undergoes many changes. Clinical studies have identified a differentiation shift in the hematopoietic system among the elderly, characterized by an increased proportion of myeloid progenitor cells and a decreased proportion of lymphoid progenitor cells (Bowen et al., 2019). Red blood cells, hemoglobin and platelets tend to be lower in healthy elderly men than in young men, and anemia occurs in the elderly. The experiment indicated a tendency for increased RBC, HGB, and PLT levels in the peripheral blood of naturally aging rats, differing somewhat from those observed in healthy elderly humans. This suggests that there may be some differences in platelet number and function compensation between aged rats and the elderly. However, there are other studies that show that aging is also manifested by bone marrow and platelet skewing, inflammation, and expansion of clonal hematopoiesis. The inflammatory state encourages a constant differentiation of hematopoietic stem cells towards megakaryocytes, which ultimately leads to an elevated platelet count and a rise in the platelet ratio (Siddhartha, 2020). This suggests that aged rats may be in a chronic inflammatory state and may also imply that aged rats may suffer from senile polycythemia vera (PV), which could potentially serve as an animal model for PV. The results showed that BZU significantly reduced whole blood viscosity and lowered HGB and PLT levels. This suggests that it may have an ameliorative effect on thrombosis and hematopoiesis and may have therapeutic effects on youth-related blood disorders.

In our investigation utilizing UPLC-EIS-MS, we identified 69 active compounds within BZU. Through network pharmacological analysis of BZ’s anti-aging properties, 37 nodes were identified, including key targets such as AKT1, TNF, PTEN, FOS, and PARP1. GO and KEGG analyses indicate that the PI3K/AKT is an upstream signaling pathway capable of regulating NF-κB, both of which are crucial in combating immunosenescence. As one of the crucial kinases involved in T-cell priming signal transduction, PI3K plays a role in anti-apoptosis and promoting cell proliferation and differentiation. It is associated with cell survival, tumorigenesis, and apoptosis, and holds significant importance in the phosphorylation of Akt. Studies have indicated that during the aging process, the PI3K pathway becomes overactivated. In particular, the dominant activating mutation of PI3K leads to T-cell dysfunction and immunosenescence (Carrie et al., 2013). When PI3K is activated, it generates phosphatidylinositol-3,4,5-trisphosphate (PIP3). PIP3 binds to the intracellular signaling protein PDK1, thereby activating AKT. The kinase PDK1 mediates the regulation of the PI3K/AKT signaling pathway, which is involved in the regulation of immune cell development as well as T cell subpopulation differentiation (Zhen et al., 2021). Subsequently, activated PI3K and AKT participate in the phosphorylation and nuclear translocation of NF-κ B, promoting the production of inflammatory mediators, leading to an imbalance in the inflammatory regulatory network of the immune system, and ultimately leading to inflammatory aging of the body (Claudio et al., 2018).

We initially investigated BZU’s impact on the differentiation of peripheral blood immune cells, including leukocytes, B, NK, and T cells subsets. Recent research indicates a strong correlation between immune function decline and aging (Anna et al., 2019; Liying and Tao, 2020; Zhong et al., 2020). In 1969, Professor Walfod proposed the doctrine of immunosenescence, which considers that the senescence of immune cells and the aging of immune organs are the essential causes of organismal aging (Walford, 1969). It is well known that the level of WBC is an indicator of immune function. Moreover, research has indicated that the modulation of white blood cell levels can prevent chronic diseases and alleviate the burden of aging (Gurum et al., 2020). Studies have also found that three dose groups of BZ water extract can significantly reduce the aging performance score and the number of spontaneous activities of naturally aging mice to a certain extent. Additionally, it can increase the number of whole blood WBC, RBC, HGB, and lymphocytes, regulate T-cell ratios, and improve age-related functional declines such as immunosuppression, insufficient hematopoiesis, and memory attenuation in naturally aging mice, demonstrating significant therapeutic advantages (Qidi et al., 2021). The study demonstrated that BZU significantly increased WBC levels in naturally aging rats, indicating its potential to enhance immune function and delay aging by boosting leukocyte levels.

In addition, as the body ages, the immune system exhibits a gradual decline in response to antigens and becomes less reactive to foreign antigens. T lymphocytes are key effector cells in immune regulation and cellular immunity, crucial for organismal defense. Aging prevents the development of naïve T cells and increases memory T cells as antigen stimulation increases due to decreased development and maturation (Tae-Kyung et al., 2018). The decline of CD8^+^T cells during senescence was more pronounced than that of CD4^+^T cells (Czesnikiewicz Guzik et al., 2008). Age-related changes in regulatory T cells were observed, marked by an increase in Treg cell numbers. The results of this experiment showed that the proportions of lymphocytes and T, Th, naïve Tc, naïve Th and effector Tc cells decreased significantly in naturally aging rats showed a decreasing trend, while the proportions of Treg, Tc, memory Tc and memory Th cells increased significantly, which is consistent with the dysregulation of cellular subsets in the elderly. The administration at BZU showed an increase in lymphocytes and various T cell subpopulations (Th, naïve Tc, naïve Th, effector Tc) and a significant decrease in Tc, memory Tc and memory Th cells. It appears that BZU increases the number of T lymphocytes and improves the dysregulation of T lymphocyte subpopulations in naturally aging rats, and regulates lymphocyte function.

Numerous studies indicate that aging alters Th1 and Th2 cell counts, disrupting the Th1/Th2 homeostatic balance (Huang et al., 2013). Transcriptome sequencing revealed significant upregulation of inflammation-related genes and signaling pathways in aging tissues (Ahadi et al., 2020). Simultaneously, the elderly exhibit elevated blood levels of pro-inflammatory factors, including CRP, IL-6, and TNF-α. Inhibition of the PI3K/Akt signaling pathway reduced TNF-α-induced NP cell senescence (Li et al., 2017). This study found that aging rats exhibited significantly reduced serum levels of IL-2, IL-4, and IL-10, alongside significantly increased serum levels of IL-6, IFN-γ, and TNF-α. After administration of BZU, serum levels of IL-2 and IL-4 significantly increased, whereas IL-6, IFN-γ, and TNF-α levels significantly decreased. This indicates that BZU can alter the levels of immune molecules in the aging body, inhibit inflammatory responses, and improve Th1 and Th2 cell imbalance.

These studies suggest that BZU regulates the development and differentiation of peripheral blood immune cells. After that, we examined the impact of BZU on spleen pathology and immune cell proliferation. BZU significantly ameliorated aging rats’ spleen histopathology in HE studies. T and B lymphocytes, the primary cells of the immune system, can be activated by Con A and LPS, respectively. Their proliferative activity influences the level of immune response and is a common indicator of immune competence. It has been shown that the proliferative capacity of lymphocytes is significantly reduced in 7-month-old mice compared to 2-month-old young C57BL/6J mice, and the proliferative capacity decreases with age (Jing et al., 2017). Studies have indicated that BZ granules, BZ polysaccharides, Atractylenolide I, and Atractylenolide III can effectively promote the proliferation of splenic lymphocytes in normal mice within a concentration range of 0.2–125 μg/mL (Cairan, 2016). Other research has found that water extracts of BZ can enhance the immune response of mice to foot-and-mouth disease vaccination, thereby increasing the proliferation rate of splenic lymphocytes (Ruili et al., 2009). The experiment demonstrated that naturally aging rats exhibited a significant reduction in the proliferative capacity of T and B lymphocytes, whereas BZU administration notably enhanced this capacity.

The above results indicate that BZU has a modulating effect on the differentiation and proliferation of immune cells and T-cell subpopulations in the peripheral blood and spleen of naturally aging rats and can balance the levels of immune molecules. Network pharmacology enrichment analysis identified the PI3K/AKT/NF-κB signaling pathway as crucial. According to reports, pterostilbene alleviates chondrocyte senescence induced by IL-1β through inhibiting the PI3K/AKT/NF-κB signaling pathway (Linjie et al., 2021). Other studies have found that ginsenoside Rk1 can inhibit the PI3K/AKT/NF-κB signaling pathway both *in vitro* and *in vivo*, regulate the secretion of various proinflammatory cytokines, alleviate ultraviolet-induced inflammatory responses, and improve skin aging caused by ultraviolet radiation (Yannan et al., 2022). Consequently, we employed quantitative RT-PCR, Western blotting, and immunohistochemistry to examine protein and mRNA expression within this pathway. The results of this experiment were consistent with the fact that, after aging, the expression level of CD3 protein decreased but activated the PI3K/AKT pathway. Significant increases in PI3K and NF-κB protein levels were observed, while IKK/α/β protein expression levels were seen to increase as well. The AM group showed significantly elevated mRNA expression levels of *Pik3cg*, *Akt1*, *Pdk1,* and *Nfκb1* compared to the AC group. AKT activates the NF-κB signaling pathway by reducing IκB-mediated inhibition, leading to increased expression of NF-κB proteins and mRNAs in the nucleus. After BZU administration, CD3 protein expression was significantly upregulated, and PI3K, p-AKT, IKKα/β, and NF-κB protein levels were significantly downregulated. Furthermore, mRNA expression levels of *Pik3cg*, *Akt1*, *Pdk1*, and *Nf*κ*b1* significantly decreased.

In summary, our study suggests that BZU may boost lymphocyte proliferation by inhibiting the PI3K/Akt/NF-κB signaling pathway, correcting immune cell imbalances, reducing inflammatory responses, and ultimately enhancing immune function and potentially delaying aging. This discovery provides a comprehensive theoretical basis for future research on BZ’s improvement of immunosenescence. However, it's worth noting that this study did not explore the main pharmacologically active components in BZU, which represents a limitation of this research and an area worthy of further exploration in future studies. Meanwhile, in terms of animal model selection, it is necessary to include animals from multiple age groups to determine whether the drug has a specific age-independent immunomodulatory effect on elderly mice, which also requires additional research. This study demonstrates that BZ exhibits significant efficacy in enhancing immunity and delaying aging, providing a certain basis for the development, application, and clinical use of BZ-related anti-aging products.

## Data Availability

The original contributions presented in the study are included in the article/supplementary material, further inquiries can be directed to the corresponding authors.

## Abbreviations

AKT, protein kinase B; BP, biological process; BZ, Atractylodis macrocephalae rhizoma; BZU, BZ ultrafine powder; CC, Cellular component; GO, Gene ontology; HE, Hematoxylin-eosin; HGB, hemoglobin; HPLC, High-performance liquid chromatography; IFN-γ, Interferon-γ; IL-2, Interleukin 2; IL-4, Interleukin 4; IL-6, Interleukin 6; IL-10, Interleukin 10; KEGG, Kyoto encyclopedia of genomes; MF, molecular function; PDK1, phosphatidylkinase-dependent kinase 1; PI3K, phosphatidylinositol 3-kinase; PLT, blood platelet; PPI, Protein-protein interaction; RBC, Red blood cell; SEM, Scanning electron microscope; Tc, CD8^+^, Cytotoxic T cells; Th, CD4^+^, T helper cells; Th1, Helper T cells 1; Th2, Helper T cells 2; TNF-α, Tumor necrosis factor-α; Treg, CD4^+^CD25^+^Foxp3^+^; Regulatory T cells; WBC, white blood cell.
