# Supplementary material for: The ultrafine powder of atractylodis macrocephalae rhizoma improves immune function in naturally aging rats by regulating the PI3K/Akt/NF-κB signaling pathway
Source: Front Pharmacol. 2025 Apr 4;16:1550357. doi: 10.3389/fphar.2025.1550357 (PMC12006087; doi:10.3389/fphar.2025.1550357)
Supplement: Supplementary file 2 [file Table2.docx]

Supplementary Table S2 Known therapeutic drug targets for the treatment of aging.

| **NO.** | **Key words** | **Protein names** | **Uniprot** | **Geng names** |
| --- | --- | --- | --- | --- |
| 1 | AGING | Adenosine receptor A3 | P0DMS8 | ADORA3 |
| 2 | AGING | Adenosine deaminase | P00813 | ADA |
| 3 | AGING | Adenosine kinase | P55263 | ADK |
| 4 | AGING | Adenosine receptor A1 | P30542 | ADORA1 |
| 5 | AGING | Adenosine receptor A2a | P29274 | ADORA2A |
| 6 | AGING | Adenosine receptor A2b | P29275 | ADORA2B |
| 7 | AGING | Solute carrier family 28 member 3 | Q9HAS3 | SLC28A3 |
| 8 | AGING | Equilibrative nucleoside transporter 1 | Q99808 | SLC29A1 |
| 9 | AGING | Equilibrative nucleoside transporter 2 | Q14542 | SLC29A2 |
| 10 | AGING | Equilibrative nucleoside transporter 3 | Q9BZD2 | SLC29A3 |
| 11 | AGING | Equilibrative nucleoside transporter 4 | Q7RTT9 | SLC29A4 |
| 12 | AGING | Sodium/nucleoside cotransporter 1 | O00337 | SLC28A1 |
| 13 | AGING | Sodium/nucleoside cotransporter 2 | O43868 | SLC28A2 |
| 14 | AGING | Gap junction alpha-1 protein | P17302 | GJA1 |
| 15 | AGING | Adenylate kinase isoenzyme 1 | P00568 | AK1 |
| 16 | AGING | Nucleoside diphosphate kinase A | P15531 | NME1 |
| 17 | AGING | Nucleoside diphosphate kinase B | P22392 | NME2 |
| 18 | AGING | Purine nucleoside phosphorylase | P00491 | PNP |
| 19 | AGING | Xanthine dehydrogenase/oxidase | P47989 | XDH |
| 20 | AGING | Serum albumin | P02768 | ALB |
| 21 | AGING | Brain Cytoplasmic RNA 1 | I7G5M5 | BCYRN1 |
| 22 | AGING | KCNQ1 Downstream Neighbor | Q9H478 | KCNQ1DN |
| 23 | AGING | Platelet Derived Growth Factor Receptor Beta | P09619 | PDGFRB |
| 24 | AGING | Lamin A/C | P02545 | LMNA |
| 25 | AGING | Tumor Protein P63 | Q9H3D4 | TP63 |
| 26 | AGING | Regucalcin | Q15493 | RGN |
| 27 | AGING | AKT Serine/Threonine Kinase 1 | P31749 | AKT1 |
| 28 | AGING | Serpin Family E Member 1 | P05121 | SERPINE1 |
| 29 | AGING | Tumor Protein P53 | P04637 | TP53 |
| 30 | AGING | Mitogen-Activated Protein Kinase 1 | P28482 | MAPK1 |
| 31 | AGING | Jun Proto-Oncogene, AP-1 Transcription Factor Subunit | P05412 | JUN |
| 32 | AGING | Peroxisome Proliferator Activated Receptor Gamma | P37231 | PPARG |
| 33 | AGING | Matrix Metallopeptidase 7 | P09237 | MMP7 |
| 34 | AGING | Hypoxia Inducible Factor 1 Subunit Alpha | Q16665 | HIF1A |
| 35 | AGING | Superoxide Dismutase 1 | P00441 | SOD1 |
| 36 | AGING | Transforming Growth Factor Beta Receptor 2 | P37173 | TGFBR2 |
| 37 | AGING | Signal Transducer And Activator Of Transcription 3 | P40763 | STAT3 |
| 38 | AGING | Sirtuin 1 | Q96EB6 | SIRT1 |
| 39 | AGING | Superoxide Dismutase 2 | P04179 | SOD2 |
| 40 | AGING | RE1 Silencing Transcription Factor | Q13127 | REST |
| 41 | AGING | Forkhead Box O3 | O43524 | FOXO3 |
| 42 | AGING | Angiotensinogen | P01019 | AGT |
| 43 | AGING | Endothelin 1 | P05305 | EDN1 |
| 44 | AGING | Opioid receptor sigma 1 | Q99720 | SIGMAR1 |
| 45 | AGING | Adrenergic receptor beta-2 | P07550 | ADRB2 |
| 46 | AGING | Clusterin | P10909 | CLU |
| 47 | AGING | AMYLOID BETA A4 PRECURSOR PROTEIN | P05067 | APP |
| 48 | AGING | S100 CALCIUM-BINDING PROTEIN A16 | Q96FQ6 | S100A16 |
| 49 | AGING | POLYMERASE, DNA, GAMMA | P54098 | POLG |
| 50 | AGING | CYCLIN-DEPENDENT KINASE INHIBITOR 2A | P42771 | CDKN2A |
| 51 | AGING | WERNER SYNDROME | Q14191 | WRN |
| 52 | AGING | INSULIN-LIKE GROWTH FACTOR I | P05019 | IGF1 |
| 53 | AGING | NUCLEAR FACTOR KAPPA-B, SUBUNIT 1 | P19838 | NFKB1 |
| 54 | AGING | EXCISION REPAIR, COMPLEMENTING DEFECTIVE, IN CHINESE HAMSTER, 4 | Q92889 | ERCC4 |
| 55 | AGING | KLOTHO | Q9UEF7 | KL |
| 56 | AGING | COLLAGEN, TYPE XVII, ALPHA-1 | Q9UMD9 | COL17A1 |
| 57 | AGING | TELOMERASE REVERSE TRANSCRIPTASE | O14746 | TERT |
| 58 | AGING | TOLL-LIKE RECEPTOR 2 | O60603 | TLR2 |
| 59 | AGING | INHIBITOR OF NUCLEAR FACTOR KAPPA-B KINASE, SUBUNIT BETA | O14920 | IKBKB |
| 60 | AGING | GONADOTROPIN-RELEASING HORMONE 1 | P01148 | GNRH1 |
| 61 | AGING | SOLUTE CARRIER FAMILY 4 (ANION EXCHANGER), MEMBER 1 | P02730 | SLC4A1 |
| 62 | AGING | BLOOM SYNDROME | P54132 | BLM |
| 63 | AGING | FORKHEAD BOX O1A | Q12778 | FOXO1A |
| 64 | AGING | ZINC METALLOPROTEINASE STE24 | O75844 | ZMPSTE24 |
| 65 | AGING | CELL DIVISION CYCLE 42 | P60953 | CDC42 |
| 66 | AGING | SUCCINATE DEHYDROGENASE COMPLEX, SUBUNIT C, INTEGRAL MEMBRANE PROTEIN, 15-KD | Q99643 | SDHC |
| 67 | AGING | INSULIN-LIKE GROWTH FACTOR I RECEPTOR | P08069 | IGF1R |
| 68 | AGING | WINGLESS-TYPE MMTV INTEGRATION SITE FAMILY, MEMBER 5A | P41221 | WNT5A |
| 69 | AGING | SIRTUIN 6 | Q8N6T7 | SIRT6 |
| 70 | AGING | EXCISION REPAIR, COMPLEMENTING DEFECTIVE, IN CHINESE HAMSTER, 1 | P07992 | ERCC1 |
| 71 | AGING | HEAT-SHOCK TRANSCRIPTION FACTOR 1 | Q00613 | HSF1 |
| 72 | AGING | WINGLESS-TYPE MMTV INTEGRATION SITE FAMILY, MEMBER 3A | P56704 | WNT3A |
| 73 | AGING | MYOTUBULARIN-RELATED PROTEIN 14 | Q8NCE2 | MTMR14 |
| 74 | AGING | PEROXISOME PROLIFERATOR-ACTIVATED RECEPTOR-ALPHA | Q07869 | PPARA |
| 75 | AGING | MEMBRANE METALLOENDOPEPTIDASE | P08473 | MME |
| 76 | AGING | TUMOR NECROSIS FACTOR RECEPTOR SUPERFAMILY, MEMBER 1A | P19438 | TNFRSF1A |
| 77 | AGING | EXCISION REPAIR, COMPLEMENTING DEFECTIVE, IN CHINESE HAMSTER, 2 | P18074 | ERCC2 |
| 78 | AGING | MYD88 INNATE IMMUNE SIGNAL TRANSDUCTION ADAPTOR | Q99836 | MYD88 |
| 79 | AGING | BROMODOMAIN ADJACENT TO ZINC FINGER DOMAIN, 2B | Q9UIF8 | BAZ2B |
| 80 | AGING | TOLL-LIKE RECEPTOR 4 | O00206 | TLR4 |
| 81 | AGING | EUCHROMATIC HISTONE METHYLTRANSFERASE 1 | Q9H9B1 | EHMT1 |
| 82 | AGING | FORKHEAD BOX O4 | P98177 | FOXO4 |
| 83 | AGING | XPA, DNA DAMAGE RECOGNITION AND REPAIR FACTOR | P23025 | XPA |
| 84 | AGING | DIABETES AND DEAFNESS, MATERNALLY INHERITED | Q8VYU8 | MIDD |
| 85 | AGING | HUNTINGTON DISEASE | P42858 | HD |
| 86 | AGING | CEREBRORETINAL MICROANGIOPATHY WITH CALCIFICATIONS AND CYSTS 2 | Q9H668 | CRMCC2 |
| 87 | AGING | ADENYLATE CYCLASE-ACTIVATING POLYPEPTIDE 1 | P18509 | ADCYAP1 |
| 88 | AGING | APOLIPOPROTEIN E | P02649 | APOE |
| 89 | AGING | LAMININ, BETA-3 | Q13751 | LAMB3 |
| 90 | AGING | G PROTEIN-COUPLED RECEPTOR 3 | P46089 | GPR3 |
| 91 | AGING | TRANSFORMING GROWTH FACTOR, BETA-1 | P01137 | TGFB1 |
| 92 | AGING | DREBRIN E | Q16643 | DBN1 |
| 93 | AGING | EXCISION REPAIR, COMPLEMENTING DEFECTIVE, IN CHINESE HAMSTER, 5 | P28715 | ERCC5 |
| 94 | AGING | CHEMOKINE, CC MOTIF, LIGAND 11 | P51671 | CCL11 |
| 95 | AGING | ATR SERINE/THREONINE KINASE | Q13535 | ATR |
| 96 | AGING | PEPTIDE METHIONINE SULFOXIDE REDUCTASE | Q9UJ68 | MSRA |
| 97 | AGING | PEROXISOME PROLIFERATOR-ACTIVATED RECEPTOR-GAMMA, COACTIVATOR 1, ALPHA | Q9UBK2 | PPARGC1A |
| 98 | AGING | GHRELIN | Q9UBU3 | GHRL |
| 99 | AGING | ARYL HYDROCARBON RECEPTOR NUCLEAR TRANSLOCATOR-LIKE PROTEIN | O00327 | ARNTL |
| 100 | AGING | PROTEIN PHOSPHATASE 1, REGULATORY SUBUNIT 10 | Q96QC0 | PPP1R10 |
| 101 | AGING | RECQ PROTEIN-LIKE 4 | O94761 | RECQL4 |
| 102 | AGING | GROWTH/DIFFERENTIATION FACTOR 11 | O95390 | GDF11 |
| 103 | AGING | NLR FAMILY, PYRIN DOMAIN-CONTAINING 3 | Q96P20 | NLRP3 |
| 104 | AGING | PIEZO-TYPE MECHANOSENSITIVE ION CHANNEL COMPONENT 1 | Q92508 | PIEZO1 |
| 105 | AGING | CDGSH IRON SULFUR DOMAIN PROTEIN 2 | Q8N5K1 | CISD2 |
| 106 | AGING | B-CELL CLL/LYMPHOMA 2 | P10415 | BCL2 |
| 107 | AGING | CD22 ANTIGEN | P20273 | CD22 |
| 108 | AGING | APOLIPOPROTEIN C-I | P02654 | APOC1 |
| 109 | AGING | CHOLINERGIC RECEPTOR, NEURONAL NICOTINIC, BETA POLYPEPTIDE 2 | P17787 | CHRNB2 |
| 110 | AGING | MATRIX METALLOPROTEINASE 1 | P03956 | MMP1 |
| 111 | AGING | POLY(ADP-RIBOSE) POLYMERASE 1 | P09874 | PARP1 |
| 112 | AGING | FORKHEAD BOX M1 | Q08050 | FOXM1 |
| 113 | AGING | CHEMOKINE, CXC MOTIF, LIGAND 12 | P48061 | CXCL12 |
| 114 | AGING | INTEGRIN, BETA-4 | P16144 | ITGB4 |
| 115 | AGING | LINE RETROTRANSPOSABLE ELEMENT 1 | Q9UN81 | LRE1 |
| 116 | AGING | SYNUCLEIN, ALPHA | P37840 | SNCA |
| 117 | AGING | HETEROGENEOUS NUCLEAR RIBONUCLEOPROTEIN A1 | P09651 | HNRNPA1 |
| 118 | AGING | HETEROGENEOUS NUCLEAR RIBONUCLEOPROTEIN A2/B1 | P22626 | HNRNPA2B1 |
| 119 | AGING | HEAT-SHOCK 70-KD PROTEIN 9 | P38646 | HSPA9 |
| 120 | AGING | 11-BETA-HYDROXYSTEROID DEHYDROGENASE, TYPE I | P28845 | HSD11B1 |
| 121 | AGING | PROHIBITIN | P35232 | PHB |
| 122 | AGING | RETINOID ISOMEROHYDROLASE RPE65 | Q16518 | RPE65 |
| 123 | AGING | NOTCH RECEPTOR 1 | P46531 | NOTCH1 |
| 124 | AGING | TRYPTOPHAN HYDROXYLASE 1 | P17752 | TPH1 |
| 125 | AGING | UBIQUITIN B | P0CG47 | UBB |
| 126 | AGING | ESTROGEN RECEPTOR 1 | P03372 | ESR1 |
| 127 | AGING | GRANULIN PRECURSOR | P28799 | GRN |
| 128 | AGING | TELOMERIC REPEAT-BINDING FACTOR 1 | P54274 | TERF1 |
| 129 | AGING | PROP PAIRED-LIKE HOMEOBOX 1 | O75360 | PROP1 |
| 130 | AGING | EGF-CONTAINING FIBULIN-LIKE EXTRACELLULAR MATRIX PROTEIN 1 | Q12805 | EFEMP1 |
| 131 | AGING | ENHANCER OF ZESTE 2 POLYCOMB REPRESSIVE COMPLEX 2 SUBUNIT | Q15910 | EZH2 |
| 132 | AGING | COENZYME Q7, HYDROXYLASE | Q99807 | COQ7 |
| 133 | AGING | BASONUCLIN 1 | Q01954 | BNC1 |
| 134 | AGING | TELOMERIC REPEAT-BINDING FACTOR 2 | Q15554 | TERF2 |
| 135 | AGING | DOLICHYL-DIPHOSPHOOLIGOSACCHARIDE-PROTEIN GLYCOSYLTRANSFERASE | P39656 | DDOST |
| 136 | AGING | EUKARYOTIC TRANSLATION INITIATION FACTOR 4E-BINDING PROTEIN 1 | Q13541 | EIF4EBP1 |
| 137 | AGING | FORKHEAD BOX A1 | P55317 | FOXA1 |
| 138 | AGING | SERINE/THREONINE PROTEIN KINASE 4 | Q13043 | STK4 |
| 139 | AGING | PROTEIN PHOSPHATASE, MAGNESIUM/MANGANESE-DEPENDENT, 1D | O15297 | PPM1D |
| 140 | AGING | 3-PHOSPHOINOSITIDE-DEPENDENT PROTEIN KINASE 1 | O15530 | PDPK1 |
| 141 | AGING | BTB AND CNC HOMOLOGY 1 | O14867 | BACH1 |
| 142 | AGING | DNA METHYLTRANSFERASE 3A | Q9Y6K1 | DNMT3A |
| 143 | AGING | HISTONE GENE CLUSTER 1, H3 HISTONE FAMILY, MEMBER A | P68431 | HIST1H3A |
| 144 | AGING | HISTONE GENE CLUSTER 1, H4 HISTONE FAMILY, MEMBER A | P62805 | HIST1H4A |
| 145 | AGING | PATATIN-LIKE PHOSPHOLIPASE DOMAIN-CONTAINING PROTEIN 6 | Q8IY17 | PNPLA6 |
| 146 | AGING | POTASSIUM CHANNEL, VOLTAGE-GATED, ISK-RELATED SUBFAMILY, MEMBER 2 | Q9Y6J6 | KCNE2 |
| 147 | AGING | NLR FAMILY, PYRIN DOMAIN-CONTAINING 1 | Q9C000 | NLRP1 |
| 148 | AGING | CELL DIVISION CYCLE AND APOPTOSIS REGULATOR 2 | Q8N163 | CCAR2 |
| 149 | AGING | POTASSIUM CHANNEL, VOLTAGE-GATED, KQT-LIKE SUBFAMILY, MEMBER 1 | P51787 | KCNQ1 |
| 150 | AGING | ATM SERINE/THREONINE KINASE | Q13315 | ATM |
| 151 | AGING | CLN5 INTRACELLULAR TRAFFICKING PROTEIN | O75503 | CLN5 |
| 152 | AGING | WERNER HELICASE-INTERACTING PROTEIN 1 | Q96S55 | WRNIP1 |
| 153 | AGING | VON HIPPEL-LINDAU TUMOR SUPPRESSOR | P40337 | VHL |
| 154 | AGING | PEROXISOME PROLIFERATOR-ACTIVATED RECEPTOR-GAMMA, COACTIVATOR 1, BETA | Q86YN6 | PPARGC1B |
| 155 | AGING | WW, C2, AND COILED-COIL DOMAIN-CONTAINING 1 | Q8IX03 | WWC1 |
| 156 | AGING | COILED-COIL-HELIX-COILED-COIL-HELIX DOMAIN-CONTAINING PROTEIN 2 | Q9Y6H1 | CHCHD2 |
| 157 | AGING | PR DOMAIN-CONTAINING PROTEIN 13 | Q9H4Q3 | PRDM13 |
| 158 | AGING | INSULIN RECEPTOR SUBSTRATE 1 | P35568 | IRS1 |
| 159 | AGING | PARAOXONASE 1 | P27169 | PON1 |
| 160 | AGING | POLYCYSTIC KIDNEY DISEASE 1 WITH OR WITHOUT POLYCYSTIC LIVER DISEASE | P98161 | PKD1 |
| 161 | AGING | CHOREOACANTHOCYTOSIS | Q96RL7 | CHAC |
| 162 | SENESCENCE | MORTALITY FACTOR 4 | Q15014 | MORF4L2 |
| 163 | SENESCENCE | MORTALITY FACTOR 4 | Q9UBU8 | MORF4L1 |
| 164 | SENESCENCE | CLAUDIN 1 | O95832 | CLDN1 |
| 165 | SENESCENCE | ATPase 13A3 | Q9H7F0 | ATP13A3 |
| 166 | SENESCENCE | LEO1 HOMOLOG, PAF1/RNA POLYMERASE II COMPLEX COMPONENT | Q8WVC0 | LEO1 |
| 167 | SENESCENCE | RIBOSOMAL L1 DOMAIN-CONTAINING PROTEIN 1 | O76021 | RSL1D1 |
| 168 | SENESCENCE | B-RAF PROTOONCOGENE, SERINE/THREONINE KINASE | P15056 | BRAF |
| 169 | SENESCENCE | CYTOPLASMIC POLYADENYLATION ELEMENT-BINDING PROTEIN 1 | Q9BZB8 | CPEB1 |
| 170 | SENESCENCE | HRAS PROTOONCOGENE, GTPase | P01112 | HRAS |
| 171 | SENESCENCE | KRAS PROTOONCOGENE, GTPase | P01116 | KRAS |
| 172 | SENESCENCE | INTERFERON, GAMMA | P01579 | IFNG |
| 173 | SENESCENCE | SIGNAL TRANSDUCER AND ACTIVATOR OF TRANSCRIPTION 1 | P42224 | STAT1 |
| 174 | SENESCENCE | TUMOR NECROSIS FACTOR | P01375 | TNF |
| 175 | SENESCENCE | MITOGEN-ACTIVATED PROTEIN KINASE-ACTIVATED PROTEIN KINASE 5 | Q8IW41 | MAPKAPK5 |
| 176 | SENESCENCE | BASIC HELIX-LOOP-HELIX FAMILY, MEMBER E40 | O14503 | BHLHE40 |
| 177 | SENESCENCE | F-BOX ONLY PROTEIN 31 | Q5XUX0 | FBXO31 |
| 178 | SENESCENCE | CYCLIN-DEPENDENT KINASE INHIBITOR 1A | P38936 | CDKN1A |
| 179 | SENESCENCE | ACTIVATING TRANSCRIPTION FACTOR 4 | P18848 | ATF4 |
| 180 | SENESCENCE | GATA-BINDING PROTEIN 4 | P43694 | GATA4 |
| 181 | SENESCENCE | CYCLIN-DEPENDENT KINASE INHIBITOR 1B | P46527 | CDKN1B |
| 182 | SENESCENCE | FIBROBLAST GROWTH FACTOR RECEPTOR 2 | P21802 | FGFR2 |
| 183 | SENESCENCE | S-PHASE KINASE-ASSOCIATED PROTEIN 2 | Q13309 | SKP2 |
| 184 | SENESCENCE | PHOSPHATASE AND TENSIN HOMOLOG | P60484 | PTEN |
| 185 | SENESCENCE | SMAD-SPECIFIC E3 UBIQUITIN PROTEIN LIGASE 2 | Q9HAU4 | SMURF2 |
| 186 | SENESCENCE | ACUTE PROMYELOCYTIC LEUKEMIA, INDUCER OF | P29590 | PML |
| 187 | SENESCENCE | TRIPEPTIDYL PEPTIDASE II | P29144 | TPP2 |
| 188 | SENESCENCE | TRANSFORMING GROWTH FACTOR, BETA-1-INDUCED 1 | O43294 | TGFB1I1 |
| 189 | SENESCENCE | SECRETED FRIZZLED-RELATED PROTEIN 1 | Q8N474 | SFRP1 |
| 190 | SENESCENCE | MALIC ENZYME 1 | P48163 | ME1 |
| 191 | SENESCENCE | MALIC ENZYME 2 | P23368 | ME2 |
| 192 | SENESCENCE | MITOGEN-ACTIVATED PROTEIN KINASE 14 | Q16539 | MAPK14 |
| 193 | SENESCENCE | TRANSCRIPTION FACTOR A, MITOCHONDRIAL | Q00059 | TFAM |
| 194 | SENESCENCE | TOPOISOMERASE, DNA, I | P11387 | TOP1 |
| 195 | SENESCENCE | LYSYL OXIDASE-LIKE 2 | Q9Y4K0 | LOXL2 |
| 196 | SENESCENCE | DYSKERIN | O60832 | DKC1 |
| 197 | SENESCENCE | PYRUVATE DEHYDROGENASE, ALPHA-1 | P08559 | PDHA1 |
| 198 | SENESCENCE | REGULATOR OF TELOMERE ELONGATION HELICASE 1 | Q9NZ71 | RTEL1 |
| 199 | SENESCENCE | CYCLIN-DEPENDENT KINASE INHIBITOR 2A-INTERACTING PROTEIN | Q9NXV6 | CDKN2AIP |
| 200 | SENESCENCE | HMG-BOX TRANSCRIPTION FACTOR 1 | O60381 | HBP1 |
| 201 | SENESCENCE | CELLULAR REPRESSOR OF E1A-STIMULATED GENES 1 | O75629 | CREG1 |
| 202 | SENESCENCE | CYCLIN-DEPENDENT KINASE 4 | P11802 | CDK4 |
| 203 | SENESCENCE | CHEMOKINE, CXC MOTIF, LIGAND 1 | P09341 | CXCL1 |
| 204 | SENESCENCE | INHIBITOR OF DNA BINDING 1 | P41134 | ID1 |
| 205 | SENESCENCE | NUCLEAR FACTOR OF ACTIVATED T CELLS, CYTOPLASMIC, CALCINEURIN-DEPENDENT 1 | O95644 | NFATC1 |
| 206 | SENESCENCE | CATHEPSIN K | P43235 | CTSK |
| 207 | SENESCENCE | PROTEIN PHOSPHATASE 3, REGULATORY SUBUNIT B, ALPHA | P63098 | PPP3R1 |
| 208 | SENESCENCE | LIGASE IV, DNA, ATP-DEPENDENT | P49917 | LIG4 |
| 209 | SENESCENCE | SPECIAL AT-RICH SEQUENCE-BINDING PROTEIN 1 | Q01826 | SATB1 |
| 210 | SENESCENCE | ZINC FINGER- AND BTB DOMAIN-CONTAINING PROTEIN 7A | O95365 | ZBTB7A |
| 211 | SENESCENCE | ACTIVATING TRANSCRIPTION FACTOR 3 | P18847 | ATF3 |
| 212 | SENESCENCE | CYCLIN-DEPENDENT KINASE 6 | Q00534 | CDK6 |
| 213 | SENESCENCE | SOLUTE CARRIER FAMILY 13 (SODIUM-DEPENDENT DICARBOXYLATE TRANSPORTER), MEMBER 3 | Q8WWT9 | SLC13A3 |
| 214 | SENESCENCE | SUPPRESSOR OF VARIEGATION 3-9, DROSOPHILA, HOMOLOG OF, 1 | O43463 | SUV39H1 |
| 215 | SENESCENCE | NICOTINAMIDE PHOSPHORIBOSYLTRANSFERASE | P43490 | NAMPT |
| 216 | SENESCENCE | SHUGOSHIN-LIKE 1 | Q5FBB7 | SGOL1 |
| 217 | SENESCENCE | UBINUCLEIN 1 | Q9NPG3 | UBN1 |
| 218 | SENESCENCE | CHROMODOMAIN HELICASE DNA-BINDING PROTEIN 5 | Q8TDI0 | CHD5 |
| 219 | SENESCENCE | ECRG4 AUGURIN PRECURSOR | Q9H1Z8 | ECRG4 |
| 220 | SENESCENCE | STIMULATOR OF INTERFERON RESPONSE cGAMP INTERACTOR 1 | Q86WV6 | STING1 |
| 221 | SENESCENCE | RIBONUCLEASE T2 | O00584 | RNASET2 |
| 222 | SENESCENCE | CYCLIC GMP-AMP SYNTHASE | Q8N884 | CGAS |
| 223 | SENESCENCE | RB TRANSCRIPTIONAL COREPRESSOR 1 | P06400 | RB1 |
| 224 | SENESCENCE | PHD FINGER PROTEIN 12 | Q96QT6 | PHF12 |
| 225 | SENESCENCE | LAMIN B1 | P20700 | LMNB1 |
| 226 | SENESCENCE | BMI1 PROTOONCOGENE, POLYCOMB RING FINGER | P35226 | BMI1 |
| 227 | SENESCENCE | CYCLIN D1 | P24385 | CCND1 |
| 228 | SENESCENCE | T-BOX TRANSCRIPTION FACTOR 2 | Q13207 | TBX2 |
| 229 | SENESCENCE | EWS RNA-BINDING PROTEIN 1 | Q01844 | EWSR1 |
| 230 | SENESCENCE | HES FAMILY bHLH TRANSCRIPTION FACTOR 1 | Q14469 | HES1 |
| 231 | SENESCENCE | RAS HOMOLOG ENRICHED IN BRAIN | Q15382 | RHEB |
| 232 | SENESCENCE | DNA REPLICATION HELICASE/NUCLEASE 2 | P51530 | DNA2 |
| 233 | SENESCENCE | UBIQUITIN-SPECIFIC PROTEASE 16 | Q9Y5T5 | USP16 |
| 234 | SENESCENCE | CATENIN, ALPHA-LIKE, 1 | Q9UBT7 | CTNNAL1 |
| 235 | SENESCENCE | TERMINAL NUCLEOTIDYLTRANSFERASE 4B | Q8NDF8 | TENT4B |
| 236 | SENESCENCE | ISOPRENYLCYSTEINE CARBOXYLMETHYLTRANSFERASE | O60725 | ICMT |
| 237 | SENESCENCE | PHOSPHATIDYLINOSITOL 3-KINASE, CATALYTIC, DELTA | O00329 | PIK3CD |
| 238 | SENESCENCE | EUKARYOTIC TRANSLATION INITIATION FACTOR 4-GAMMA, 3 | O43432 | EIF4G3 |
| 239 | SENESCENCE | HELICASE, LYMPHOID-SPECIFIC | Q9NRZ9 | HELLS |
| 240 | SENESCENCE | 24-DEHYDROCHOLESTEROL REDUCTASE | Q15392 | DHCR24 |
| 241 | SENESCENCE | STIP1 HOMOLOGOUS AND U BOX-CONTAINING PROTEIN 1 | Q9UNE7 | STUB1 |
| 242 | SENESCENCE | SRY-BOX 9 | U5XJY7 | SOX9 |
| 243 | SENESCENCE | MONOOXYGENASE, DBH-LIKE, 1 | Q6UVY6 | MOXD1 |
| 244 | SENESCENCE | ANTI-SILENCING FUNCTION 1A HISTONE CHAPERONE | Q9Y294 | ASF1A |
| 245 | SENESCENCE | EUKARYOTIC TRANSLATION ELONGATION FACTOR 1, EPSILON-1 | O43324 | EEF1E1 |
| 246 | SENESCENCE | YIPPEE-LIKE 3 | P61236 | YPEL3 |
| 247 | SENESCENCE | SOLUTE CARRIER FAMILY 25, MEMBER 46 | Q96AG3 | SLC25A46 |
| 248 | SENESCENCE | FTO ALPHA-KETOGLUTARATE-DEPENDENT DIOXYGENASE | Q9C0B1 | FTO |
| 249 | SENESCENCE | TRANSMEMBRANE PROTEASE, SERINE 11A | Q6ZMR5 | TMPRSS11A |
| 250 | SENESCENCE | UBINUCLEIN 2 | Q6ZU65 | UBN2 |
| 251 | SENESCENCE | WD REPEAT-CONTAINING PROTEIN 73 | Q6P4I2 | WDR73 |
| 252 | SENESCENCE | KINASE NONCATALYTIC C-LOBE DOMAIN-CONTAINING PROTEIN 1 | Q76NI1 | KNDC1 |
| 253 | SENESCENCE | LEM DOMAIN-CONTAINING PROTEIN 2 | Q8NC56 | LEMD2 |
| 254 | SENESCENCE | CCR4-NOT TRANSCRIPTION COMPLEX, SUBUNIT 6-LIKE | Q96LI5 | CNOT6L |
| 255 | SENESCENCE | MUSCULAR DYSTROPHY, DUCHENNE TYPE | P11532 | DMD |
| 256 | SENESCENCE | FANCONI ANEMIA, COMPLEMENTATION GROUP A | O15360 | FANCA |
| 257 | SENESCENCE | POLYMERASE (DNA-DIRECTED), DELTA 1, CATALYTIC SUBUNIT | P28340 | POLD1 |
| 258 | SENESCENCE | NUCLEAR RECEPTOR SUBFAMILY 1, GROUP D, MEMBER 1 | P20393 | NR1D1 |
| 259 | SENESCENCE | BROMODOMAIN- AND PHD FINGER-CONTAINING PROTEIN | P55201 | BRPF1 |
| 260 | SENESCENCE | X-RAY REPAIR CROSS COMPLEMENTING 4 | Q13426 | XRCC4 |
| 261 | SENESCENCE | LACTALBUMIN, ALPHA | P00709 | LALBA |
| 262 | SENESCENCE | MELANOCORTIN 1 RECEPTOR | Q01726 | MC1R |
| 263 | SENESCENCE | CHEMOKINE, CXC MOTIF, RECEPTOR 4 | P61073 | CXCR4 |
| 264 | SENESCENCE | FOS PROTOONCOGENE, AP1 TRANSCRIPTION FACTOR SUBUNIT | P01100 | FOS |
| 265 | SENESCENCE | POU DOMAIN, CLASS 1, TRANSCRIPTION FACTOR 1 | P28069 | POU1F1 |
| 266 | SENESCENCE | RUNT-RELATED TRANSCRIPTION FACTOR 2 | Q13950 | RUNX2 |
| 267 | SENESCENCE | CYCLIN-DEPENDENT KINASE INHIBITOR 2B | P42772 | CDKN2B |
| 268 | SENESCENCE | CASEIN KINASE I, ALPHA-1 | P48729 | CSNK1A1 |
| 269 | SENESCENCE | GA-BINDING PROTEIN TRANSCRIPTION FACTOR, ALPHA SUBUNIT | Q06546 | GABPA |
| 270 | SENESCENCE | CD82 ANTIGEN | P27701 | CD82 |
| 271 | SENESCENCE | CASPASE 3, APOPTOSIS-RELATED CYSTEINE PROTEASE | P42574 | CASP3 |
| 272 | SENESCENCE | TRANSGELIN | Q01995 | TAGLN |
| 273 | SENESCENCE | PROLIFERATING CELL NUCLEAR ANTIGEN | P12004 | PCNA |
| 274 | SENESCENCE | MYC PROTOONCOGENE, bHLH TRANSCRIPTION FACTOR | P01106 | MYC |
| 275 | SENESCENCE | VIMENTIN | P08670 | VIM |
| 276 | SENESCENCE | LIGASE I, DNA, ATP-DEPENDENT | P18858 | LIG1 |
| 277 | SENESCENCE | RUNT-RELATED TRANSCRIPTION FACTOR 1, TRANSLOCATED TO, 1 | Q06455 | RUNX1T1 |
| 278 | SENESCENCE | EUKARYOTIC TRANSLATION INITIATION FACTOR 4E | P06730 | EIF4E |
| 279 | SENESCENCE | HEMOGLOBIN--BETA LOCUS | P68871 | HBB |
| 280 | SENESCENCE | HOMEOBOX A9 | P31269 | HOXA9 |
| 281 | SENESCENCE | CALCIUM CHANNEL, VOLTAGE-DEPENDENT, P/Q TYPE, ALPHA-1A SUBUNIT | O00555 | CACNA1A |
| 282 | SENESCENCE | DEAD/H-BOX HELICASE 11 | Q96FC9 | DDX11 |
| 283 | SENESCENCE | MECHANISTIC TARGET OF RAPAMYCIN | P42345 | MTOR |
| 284 | SENESCENCE | MICROTUBULE-ASSOCIATED PROTEIN 1, LIGHT CHAIN 3, ALPHA | Q9H492 | MAP1LC3A |
| 285 | SENESCENCE | SERINE/THREONINE PROTEIN KINASE 11 | Q15831 | STK11 |
| 286 | SENESCENCE | NUCLEAR RECEPTOR SUBFAMILY 1, GROUP D, MEMBER 2 | Q14995 | NR1D2 |
| 287 | SENESCENCE | POLO-LIKE KINASE 4 | O00444 | PLK4 |
| 288 | SENESCENCE | NADPH OXIDASE 4 | Q9NPH5 | NOX4 |
| 289 | SENESCENCE | BUB1 MITOTIC CHECKPOINT SERINE/THREONINE KINASE B | O60566 | BUB1B |
| 290 | SENESCENCE | DNA METHYLTRANSFERASE 3B | Q9UBC3 | DNMT3B |
| 291 | SENESCENCE | NOGGIN | Q13253 | NOG |
| 292 | SENESCENCE | AT-RICH INTERACTION DOMAIN-CONTAINING PROTEIN 1A | O14497 | ARID1A |
| 293 | SENESCENCE | ELAV-LIKE RNA-BINDING PROTEIN 1 | Q15717 | ELAVL1 |
| 294 | SENESCENCE | CELL DIVISION CYCLE 14B | O60729 | CDC14B |
| 295 | SENESCENCE | SIRTUIN 7 | Q9NRC8 | SIRT7 |
| 296 | SENESCENCE | PROTECTION OF TELOMERES 1 | Q9NUX5 | POT1 |
| 297 | SENESCENCE | LOW DENSITY LIPOPROTEIN RECEPTOR | P01130 | LDLRAP1 |
| 298 | SENESCENCE | TRAF3-INTERACTING PROTEIN 2 | O43734 | TRAF3IP2 |
| 299 | ANTIAGING | BECLIN 1 | Q14457 | BECN1 |
| 300 | ANTIAGING | SIRTUIN 2 | Q8IXJ6 | SIRT2 |
| 301 | ANTIAGING | SIRTUIN 3 | Q9NTG7 | SIRT3 |
| 302 | ANTIAGING | SIRTUIN 4 | Q9Y6E7 | SIRT4 |
| 303 | ANTIAGING | SIRTUIN 5 | Q9NXA8 | SIRT5 |
